# Physiological and Genomic Characterization of *Actinotalea subterranea* sp. nov. from Oil-Degrading Methanogenic Enrichment and Reclassification of the Family *Actinotaleaceae*

**DOI:** 10.3390/microorganisms10020378

**Published:** 2022-02-06

**Authors:** Ekaterina M. Semenova, Denis S. Grouzdev, Diyana S. Sokolova, Tatiyana P. Tourova, Andrey B. Poltaraus, Natalia V. Potekhina, Polina N. Shishina, Maria A. Bolshakova, Alexander N. Avtukh, Elena A. Ianutsevich, Vera M. Tereshina, Tamara N. Nazina

**Affiliations:** 1 Winogradsky Institute of Microbiology, Research Center of Biotechnology, Russian Academy of Sciences, 119071 Moscow, Russia; semenova_inmi@mail.ru (E.M.S.); sokolovadiyana@gmail.com (D.S.S.); tptour@rambler.ru (T.P.T.); e.a.ianutsevich@gmail.com (E.A.I.); v.m.tereshina@inbox.ru (V.M.T.); 2 SciBear OU, 10115 Tallinn, Estonia; denisgrouzdev@gmail.com; 3 Engelhardt Institute of Molecular Biology, Russian Academy of Sciences, 119991 Moscow, Russia; abpolt@gmail.com; 4 Faculty of Biology, Lomonosov Moscow State University, 119991 Moscow, Russia; potekhina56@mail.ru; 5 Geological Faculty, Lomonosov Moscow State University, 119991 Moscow, Russia; shishina_p@mail.ru (P.N.S.); m.bolshakova@oilmsu.ru (M.A.B.); 6 Skryabin Institute of Biochemistry and Physiology of Microorganisms, Pushchino Scientific Center for Biological Research, Russian Academy of Sciences, Pushchino, 142290 Moscow, Russia; avtukh@rambler.ru

**Keywords:** *Actinotalea subterranea*, polyphasic taxonomy, genome analysis, *Actinotaleaceae*, oil field

## Abstract

The goal of the present work was to determine the diversity of prokaryotes involved in anaerobic oil degradation in oil fields. The composition of the anaerobic oil-degrading methanogenic enrichment obtained from an oil reservoir was determined by 16S rRNA-based survey, and the facultatively anaerobic chemoorganotrophic bacterial strain HO-Ch2^T^ was isolated and studied using polyphasic taxonomy approach and genome sequencing. The strain HO-Ch2^T^ grew optimally at 28 °C, pH 8.0, and 1–2% (*w*/*v*) NaCl. The 16S rRNA gene sequence of the strain HO-Ch2^T^ had 98.8% similarity with the sequence of *Actinotalea ferrariae* CF5-4^T^. The genomic DNA G + C content of strain HO-Ch2^T^ was 73.4%. The average nucleotide identity (ANI) and digital DNA–DNA hybridization (dDDH) values between the genome of strain HO-Ch2^T^ and *Actinotalea* genomes were 79.8–82.0% and 20.5–22.2%, respectively, i.e., below the thresholds for species delineation. Based on the phylogenomic, phenotypic, and chemotaxonomic characterization, we propose strain HO-Ch2^T^ (= VKM Ac-2850^T^ = KCTC 49656^T^) as the type strain of a new species within the genus *Actinotalea*, with the name *Actinotalea subterranea* sp. nov. Based on the phylogenomic analysis of 187 genomes of *Actinobacteria* we propose the taxonomic revision of the genera *Actinotalea* and *Pseudactinotalea* and of the family *Actinotaleaceae*. We also propose the reclassification of *Cellulomonas carbonis* as *Actinotalea carbonis* comb. nov., *Cellulomonas bogoriensis* as *Actinotalea bogoriensis* comb. nov., *Actinotalea caeni* as *Pseudactinotalea caeni* comb. nov., and the transfer of the genus *Pseudactinotalea* to the family *Ruaniaceae* of the order *Ruaniales*.

## 1. Introduction

Oil field exploitation results in the exhaustion of oil reserves, oil biodegradation, and a decrease in oil quality. Microbial anaerobic oil degradation in oil fields is considered among the processes of transformation of certified light oil to heavy oil and then into bitumen [1]. As a rule, oil reservoirs do not contain free oxygen. Under these conditions, the possible electron acceptors for microorganisms are CO_2_, sulfate, sulfur and other oxidized sulfur compounds, or iron hydroxides [2,3]. Nitrate and other nitrogen oxides do not occur in formation water. In this ecosystem, oil is the main source of organic matter.

Anaerobic production of methane from oil was originally reported by S.I. Kuznetsov [4]. A wide range of works is devoted to the study of the composition of methanogenic syntrophic associations degrading oil [5,6,7,8,9,10]. The participation of bacteria of the *Syntrophaceae* family in the syntrophic degradation of alkanes together with methanogens has been shown [11,12,13,14,15]. Although the composition of the associations differed depending on the composition of the oil, the temperature and salinity of the habitat from which they were isolated, the main functional components were oil-degrading Deltaproteobacteria or/and Firmicutes and hydrogenotrophic and acetoclastic methanogens [16,17,18]. Using metagenomic analysis in syntrophic oil-degrading communities, in addition to the known species, new uncultured bacteria such as ‘Atribacteria’ or archaea ‘*Candidatus* Methanoliparia’, ‘*Candidatus* Argoarchaeum’, and ‘*Candidatus* Syntrophoarchaeum’ were revealed [19,20,21]. Moreover, recently non-syntrophic hydrocarbon degradation by the methanogenic archaeon ‘*Candidatus* Methanoliparum’ was shown, which essentially changes our view on anaerobic oil transformation in petroleum reservoirs [22].

Members of Actinobacteria were identified in various methanogenic crude oil-degrading consortia [14,23]. In the syntrophic thermophilic methanogenic community growing on crude oil, actinobacteria ‘*Candidatus* Syntraliphaticia’ were found, probably belonging to a new class of the phylum Actinobacteria [23]. Metatranscriptomic analysis indicated their ability to carry out anaerobic alkane degradation by activation with fumarate and subsequent oxidation to CO_2_, H_2_/formate, and acetate, which were then utilized by methanogens. Aerobic hydrocarbon-oxidizing actinobacteria of the genera *Dietzia*, *Gordonia*, *Rhodococcus*, and *Mycobacterium* have been repeatedly detected in oil fields exploited with water flooding in order to maintain formational pressure [2,24]. Based on metagenomics analysis, An and co-workers [25] have shown the existence of aerobic taxa and the genes for aerobic hydrocarbon degradation in anaerobic environments containing hydrocarbons (oil fields, oil sands, and coal beds). Although the metagenomic approach makes it possible to characterize the potential activity of a microbial community in general, isolation of components of the community and determination of their physiological and genomic properties is required to elucidate their ecological role in the environment. In the current study, a methanogenic oil-degrading enrichment culture was obtained from production water of the low-temperature oil reservoir. The microbial community composition determined by sequencing of the V4 fragments of the 16S rRNA gene revealed bacteria of the phyla Bacillota, Actinomycetota, and Pseudomonadota and of methanogenic archaea of the phylum Euryarchaeota. The key fermentative bacterial strains isolated from the enrichment belonged to the genus *Actinotalea*. These data indicate the need for further research of anaerobic oil-degrading microbial communities for elucidation of their impact in oil degradation in petroleum reservoirs.

The aim of this study was elucidation of the taxonomic position of actinobacterial strains and their role in the oil degradation. Using a polyphase taxonomy approach and genome sequencing, the fermentative strain HO-Ch2^T^ isolated from the methanogenic enrichment was described as a new species *Actinotalea subterranea* sp. nov., and its potential ecophysiological function in the oil field was discussed. Comparative analysis of 187 genomes of the class *Actinomycetia* resulted in the taxonomic revision of the family *Actinotaleaceae*. Bacteria *Cellulomonas carbonis* and *Cellulomonas bogoriensis* were reclassified as member of a genus *Actinotalea* as *Actinotalea carbonis* comb. nov. and *Actinotalea bogoriensis* comb. nov., respectively. Species *Actinotalea caeni* was assigned to a genus *Pseudactinotalea* as *Pseudactinotalea caeni* comb. nov., and the genus *Pseudactinotalea* was transferred from the family *Actinotaleaceae* of the order *Cellulomonadales* to the family *Ruaniaceae* of the order *Ruaniales*.

## 2. Materials and Methods

### 2.1. Development of Methanogenic Enrichment Growing on Crude Oil

The water sample obtained from the Cheremukhovskoe heavy oil reservoir (Ritek, Nurlat, Russia) was used for isolation of an anaerobic methanogenic enrichment growing on crude oil. The oil-bearing horizons located at the depth of 890–920 m had a temperature of 20.2–21.3 °C. Oil density of surface sample was 0.932 g/cm^3^ (at 25 °C). The Cheremukhovskoe oilfield is exploited with water-flooding; production water remaining after oil separation was used for injection into the oilfield after mixing with fresh surface water. The sample of production water re-injected into the reservoir (PWRI) was collected at the well head of injection well 5600. This water sample had low redox value (Eh –28.9 mV) and total salinity 0.621 g·L^−1^; pH 7.9. Hydrocarbonate, chloride, and sulfate were the major anions, and K^+^ + Na^+^ were the major cations. Physicochemical and microbiological parameters of formation water have been reported previously [26]. The HO-5600 (HO, heavy oil) anaerobic enrichment was obtained by inoculation of the mineral salt medium (MS) containing the following (per liter distilled water): 3.0 g MgCl_2_·6H_2_O, 0.15 g CaCl_2_·2H_2_O, 0.25 g NH_4_Cl, 0.2 g KH_2_PO_4_, 0.5 g KCl, 5.0 g NaCl, 2.5 g NaHCO_3_, 0.001 g resazurin, 0.5 g Na_2_S·9H_2_O; 1 mL·l^−1^ trace elements [27]; pH 7.0–7.2; MS medium was amended with 0.5 g yeast extract and heavy oil (0.5% *v*/*v*). Argon was used as the gas phase. Cultivation was carried out in 100-mL glass vials hermetically sealed with rubber stoppers and metal caps under stationary conditions in the dark at 28 °C. In parallel, from the 5600 injection water sample an aerobic organotrophic enrichment was obtained in the medium containing the following (per liter distilled water): 5.0 g Bacto tryptone, 2.5 g yeast extract, 1.0 g glucose, and 5.0 g NaCl; pH 7.0–7.2.

### 2.2. DNA Isolation from the Oil-Degrading Enrichment, 16S rRNA Gene Amplification, and Sequencing

The methanogenic enrichment HO-5600 (20 mL culture from one glass vial) incubated for 36 weeks was filtered through membrane filters with 0.22 μm pores (Millipore, Merck, Darmstadt, Germany). After treatment with the solution containing 0.15 M NaCl and 0.1 M Na_2_EDTA (pH 8.0), the filters with biomass were used for DNA isolation. DNA was extracted from the HO-5600 enrichment using the Pure Link Microbiome DNA Purification KIT (Thermo Fisher Scientific, Waltham, MA, USA) according to the manufacturer’s recommendations. The V4 hypervariable region of the 16S rRNA gene was amplified with the specific 515f/806 primer system [28]. Using the Cleanup Standard gel extraction kit (Evrogen, Russia), 16S rRNA gene fragments were amplified on the template of DNA isolated in three replicates, which then were combined and purified by electrophoresis in 2% agarose gel. The libraries were prepared as described previously [29]. High-throughput sequencing was conducted with a MiSeq system (Illumina, San Diego, CA, USA) and MiSeq Reagent Kit v2 (500 cycles) (Illumina, San Diego, CA, USA), according to the manufacturer’s recommendations. The obtained 250-bp reads were then processed using workflows implemented the USEARCH v.10 scripts [30]. Reads were demultiplexed, trimmed to remove the primer sequences, and quality filtered. UNOISE3 [31,32] was used to generate zero radius operational taxonomic units (zOTUs). The zOTUs were assessed using default parameters in the SILVA database (SINA, https://www.arb-silva.de/aligner/, v. 1.2.11, accessed on 29 September 2021, SILVA reference database release 138.1) [33].

### 2.3. Bacterial and Archaeal Strains

Bacterial strain HO-Ch2^T^ and methanogenic strain HO-Met1 were isolated from the HO-5600 anaerobic methanogenic enrichment grown on heavy oil. The aerobic strain HO-62b1 was isolated from the aerobic organotrophic enrichment. The strains HO-Ch2^T^ and HO-62b1 were isolated from the highest dilution of respective enrichments by successive plating on the R2A medium containing per liter distilled water: 0.5 g casein hydrolyzate, 0.5 g yeast extract, 0.5 g peptone, 0.5 g glucose, 0.5 g starch, 0.3 g K_2_HPO_4_, 0.02 g MgSO_4_, 0.3 g pyruvate, 5.0 g NaCl, and 18 g agar-agar; pH 7.0–7.2 [34]. Strains were incubated at 28–30 °C. The purity of the strains was checked by microscopy of colonies and by sequencing of the 16S rRNA genes. Strain HO-Ch2^T^ was deposited at the All-Russian collection of microorganisms (VKM; Pushchino, Moscow Region, Russia) under the number VKM Ac-2850, and at the Korean Collection for Type Cultures (KCTC; Jeongeup-si, Korea) under the number KCTC 49656. Strain *Actinotalea ferrariae* CF5-4^T^ (= KCTC 29134^T^) obtained from the KCTC (Jeongeup-si, Korea) was used as a reference strain. Methanogenic strain HO-Met1 was isolated by successive transfer from the highest dilution of the methanogenic enrichment in the liquid MS medium amended with ampicillin (10 mg·l-1) and with H_2_/CO_2_ (4:1, *v*/*v*) mixture as a gas phase.

### 2.4. DNA Extraction from New Strains, 16S rRNA Gene Sequencing, and Phylogenetic Analysis

DNA for the 16S rRNA gene or genome sequencing was extracted from biomass of the strains HO-Ch2^T^ and HO-62b1 grown aerobically on the R2A medium with 2.0% (*w*/*v*) NaCl at 28 °C. Cells were harvested after 7 days of cultivation. The cetyltrimethylammonium bromide (CTAB) method [35] was used to purify DNA from cell biomass. The 1492R and 27F primers were used to amplify the 16S rRNA genes of the strains HO-Ch2T and HO-62b2 [36]. The PCR products were sequenced using the Big Dye Terminator reagent kit, v. 3.1, at the ABI Prism 3730 DNA analyzer (Applied Biosystems, Waltham, MA, USA). Analysis of the 16S rRNA gene sequences was carried out using EzBioCloud [37]. The sequences were analyzed using the maximum-likelihood, neighbor-joining, and maximum-parsimony algorithms. First, the sequences were aligned by MUSCLE [38], and a maximum-likelihood tree was constructed using the model GTR+F+I+G4 recommended by ModelFinder [39] in IQ-Tree [40]. The MEGA7 software package was used to reconstruct the neighbor-joining and maximum-parsimony trees [41]. Bootstrap values were calculated from 1000 alternative trees. The GenBank/EMBL/DDBJ accession numbers of the 16S rRNA gene sequence of strains HO-Ch2^T^, HO-62b1, and HO-Met1 are MT225794, OK336454, and MT218393, respectively.

### 2.5. Genome Sequencing and Analyses

To construct DNA libraries for strain HO-Ch2^T^, the NEBNext DNA library prep reagent kit for Illumina (New England Biolabs, Waltham, MA, USA) was used. Sequencing of genomic DNA was performed using the Illumina HiSeq 2500 platform (Illumina, Inc., San Diego, CA, USA). The quality of raw sequence reads was checked with FastQC v. 0.11.7 (http://www.bioinformatics.babraham.ac.uk/projects/fastqc/, accessed on 29 September 2021), and low-quality reads were trimmed with Trimmomatic v. 0.36 [42]. The quality-filtered reads were de novo assembled using SPAdes v. 3.13.0 [43]. The estimated completeness and contamination were evaluated using CheckM v1.0.18 [44]. Primary annotation and identification of protein-coding sequences were performed using the NCBI Prokaryotic Genome Automatic Annotation Pipeline (PGAP) [45]. Additional gene prediction and functional annotation were performed in the Rapid Annotation using Subsystems Technology (RAST) server [46]. For the comparative metabolic study, an automatic assignment of KEGG Orthology (KO) identifiers to the proteins of *Actinotalea* type strains was completed using BlastKOALA [47]. The genome of strain HO-Ch2^T^ was deposited in GenBank/EMBL/DDBJ under the accession number VTTP00000000.1.

Phylogenomic analysis of strain HO-Ch2^T^ and members of the class *Actinomycetia* was performed based on concatenated alignment of 120 single-copy phylogenetic marker genes obtained with the GTDB-Tk v. 1.0.2, [48]. The maximum likelihood phylogenomic tree was calculated using IQ-Tree [40], based on the ModelFinder recommendations [39], and the branching support was estimated using UFBoot2 [49]. MPBoot [50] and MEGA7 [41] were used to reconstruct maximum parsimony and neighbor-joining trees, respectively. DNA-DNA hybridization (dDDH) and average nucleotide identity (ANI) of genomes were determined using Genome-to-Genome Distance Calculator (GGDC) v. 2.1 [51] and FastANI v. 1.3 [52], respectively. Average amino acid identity (AAI) was calculated with CompareM 0.0.23 (https://github.com/dparks1134/CompareM, accessed on 29 September 2021) using default parameters for blastp (i.e., e-value ≤0.001, percent identity ≥30% and alignment length ≥70%). The pangenomic analysis was done based on a bioinformatic pipeline proposed [53], using anvi’o version 6.2 [54]. Using the MCL algorithm (Euclidean distance, Ward linkage), genomes were arranged according to the distribution of gene clusters.

### 2.6. Morphological and Physiological Characterization

Strains HO-Ch2^T^ and HO-62b1 were characterized using a polyphasic taxonomic approach and compared with the reference strain *Actinotalea ferrariae* CF5-4^T^. Cell morphology was studied by epifluorescence microscopy of 5-day cultures under an Axio Imager.D1 microscope (Carl Zeiss, Germany) and by scanning electron microscopy of metal-sprayed dry cells under a Camscan-S2 scanning electron microscope (Cambridge, UK) at 20 kV accelerating voltage. Cells negatively stained with 1% (*w*/*v*) phosphotungstic acid were studied also under a JEM-100C transmission electron microscope (JEOL, Tokyo, Japan) at an accelerating voltage of 80 kV. Gram staining was performed by using a Gram staining kit (Biovitrum, Saint-Petersburg, Russia) according to the manufacturer’s instructions. Growth at different temperatures (5, 10, 15, 23, 28, 37, and 42 °C) was determined in the liquid R2A medium containing 2.0% (*w*/*v*) NaCl after incubation for 5–15 days. Salinity optimum and ranges for growth were determined in the liquid R2A medium containing 0, 0.1, 0.5, 1.0, 1.5, 2, 3.0, 4.0, 5.0, 6.0, 7.0, 8.0% (*w*/*v*) NaCl for 7 days at 28 °C. The pH range for growth was determined in the Luria-Bertani (LB) medium at pH 5.2–9.0 with increments of ~0.3–0.7 pH unit, using the appropriate citrate/phosphate (pH 5.2–7.5) and Tris/HCl (pH 8.0–9.0) buffers at optimal temperature (28 °C) and 2.0% (*w*/*v*) NaCl. Growth criteria were the change in the OD_660_ of the medium, as well as microscopy. Increases of <10%, 10–50%, and >50% in the optical density (OD) at 660 nm of the liquid media after 5–15 days of growth were scored as no utilization (–), weak utilization (w), and good utilization (+), respectively. Biochemical and enzyme characteristics of strains HO-Ch2^T^, HO-62b1 and CF5-4^T^ were determined by using API 50CH, API ^®^ ZYM, and API 20E kits (Bio-Mérieux, Marcy l’Etoile, France) according to the manufacturer’s instructions. Catalase activity was determined by the standard method with H_2_O_2_. Oxidase activity was determined using the oxidase reagent (bioMérieux, Marcy l’Etoile, France). The aerobic and anaerobic utilization of carbon sources by the strains HO-Ch2^T^ and HO-62b1 was additionally tested in MS medium without Na_2_S·9H_2_O; yeast extract was replaced with 0.3 g·L^−1^ Casamino acids. Sugars, peptone, tryptone, and yeast extract were added at a concentration of 0.5% (*w*/*v*); alcohols, salts of organic acids, at 0.2% (*w*/*v*); amino acids, at 0.1–0.2% (*w*/*v*). Inoculated medium without the relevant substrate served as the control. Strains were tested for ability to use thiosulfate (3.2 g·L^−1^), nitrate (0.85 g·L^−1^), and Fe(3+) citrate (16.0 g·L^−1^) as electron acceptors for anaerobic growth with acetate (2.0 g·L^−1^). Anaerobic growth was tested by incubation in Hungate’s tubes with Ar as a gas phase at 28 °C for 2 weeks. Sulfide was measured colorimetrically [55]; nitrite was determined using the Griess reagent. Products of glucose (5.0 g·L^−1^) fermentation in MS medium were analyzed by gas chromatography as described previously [56]. Antibiotic susceptibility was estimated in duplicate by spreading bacterial suspensions on Plate Count Agar (PCA, Merck, Darmstadt, Germany) medium with 2.0% (*w*/*v*) NaCl and applying filter paper disks (BD BBL sensi-disc antimicrobial susceptibility test discs, Becton, Dickinson and Company, USA) containing ampicillin (10 µg), chloramphenicol (30 µg), penicillin (10 µg), ciprofloxacin (5 µg), erythromycin (15 µg), gentamicin (10 µg), and kanamycin (30 µg). Susceptibility results were recorded as positive at zones with diameters higher than 10 mm after incubation at 28 °C for 2 days.

### 2.7. Chemotaxonomic Characterization

For chemotaxonomic characterization, strains HO-Ch2^T^ and *Actinotalea ferrariae* CF5-4^T^ were grown in TSB at 28 °C for 5 days. The cell biomass was dried with methanol and subjected to acidic methanolysis (1.2 M HCl/MeOH, 80 °C, 45 min). The fatty acid composition was analyzed using a Maestro gas chromatograph-mass spectrometer (Interlab, Russia) as described earlier [57]. The analysis of respiratory quinones of strains HO-Ch2^T^ and *A. ferrariae* CF5-4^T^ was performed at the All-Russian Collection of Microorganisms. Isoprenoid quinones were extracted from wet cells, purified according to Collins and Jones [58] and analyzed with a LCQ Advantage MAX mass spectrometer (Thermo Finnigan, San Jose, CA, USA). Membrane lipids were analyzed as described in Supplementary Materials [59,60,61,62,63]. Peptidoglycan and sugars in the whole cell-wall of strains HO-Ch2^T^ and CF5-4^T^ were analyzed as described in Supplementary Materials [64,65].

### 2.8. Gas Chromatography

The *n*-alkanes and *iso*-alkanes content of oil were determined by gas–liquid chromatography on a Kristall 5000.1 chromatograph (Khromatek, Yoshkar-Ola, Russia) with a flame ionization detector and a ZB-FFAP 15 m capillary column as described earlier [66]. The oil samples were also analyzed by GC-MS on Agilent 5977A MSD fitted with HP-5MS (30 m × 0.25 mm i.d. × 0.25 μm film) capillary column (Agilent Technologies, Santa Clara, CA, USA). The carrier gas was helium (1 mL·min^−1^). For saturated oil components the initial column temperature was held for 3 min at 60 °C, then was increased at a rate 27 °C·min^−1^ to 180 °C and then was increased at 6 °C·min^−1^ to a final temperature of 300 °C, which was maintained for 25 min. To assess the extent of microbial oil degradation, the relative content (%) of *n*-alkanes was analyzed with relation to the control sample.

### 2.9. Nucleotide Sequence Accession Number

The library of 16S rRNA gene fragments of the HO-5600 methanogenic enrichment culture was deposited in NCBI SRA, project no. SRR17034681. The GenBank/EMBL/DDBJ accession numbers of the 16S rRNA gene sequence of strains HO-Ch2^T^, HO-62b1, and HO-Met1 are MT225794, OK336454, and MT218393, respectively. The whole-genome shotgun project of strain HO-Ch2^T^ has been deposited at DDBJ/EMBL/GenBank under the accession VTTP00000000.1. The raw FASTQ reads have been deposited in the NCBI SRA database under the accession no. SRR10092497.

## 3. Results

### 3.1. Methane Production and Phylogenetic Diversity of Prokaryotes in the Anaerobic Oil-Degrading Enrichment

**Figure 1:**
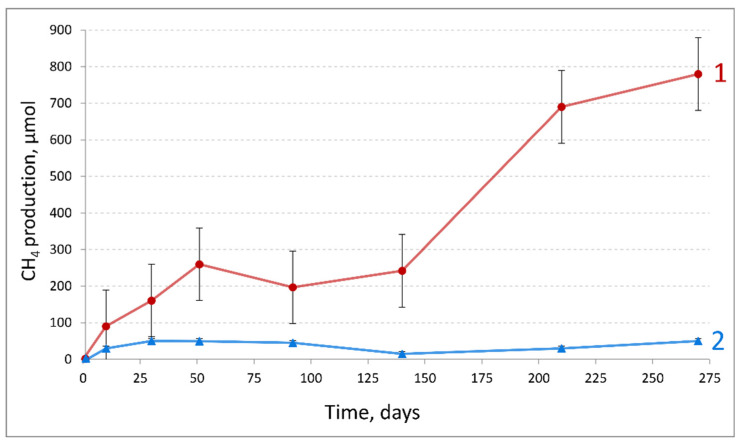
Methane formation in the HO-5600 anaerobic enrichment growing on crude oil (1) and in the control sterile medium with crude oil (2).

Dynamics of methane production by the HO-5600 oil-degrading enrichment obtained from production water of the heavy oil reservoir was monitored (Figure 1). Production of H_2_ was observed during the initial stage of incubation; it was subsequently consumed, and methane became the main gaseous product formed at the rate of 12.4 µmol·g of oil^−1^·day^−1^. After 270 days of incubation, methane concentration in the gas phase was 17,460 ppm and 80 mg·l^−1^ acetate was found in the medium of the oil-degrading enrichment. HO-5600 methanogenic enrichment utilized the narrow range of C_16_–C_19_ *n*-alkanes in crude oil (Figure S1a–c).

The composition of the HO-5600 enrichment was determined by high-throughput sequencing of the V4 region of the 16S rRNA gene after 36 weeks of incubation with crude oil. A total of 72,601 16S rRNA gene fragments were obtained for the library. The sequences were grouped into 244 zOTUs. The oil-degrading enrichment was characterized by a low prokaryotic diversity. Archaea (1% of the sequences in the library) were represented by hydrogenothrophic methanogens of the genus *Methanobacterium* of the phylum Euryarchaeota. Bacteria (99%) predominated in the enrichment and belonged to the phyla Bacillota [67] (53%, including the genus *Sedimentibacter*, 49.1%), Actinomycetota (42%, including the genera *Actinotalea*, 26.8%, and *Nocardioides*, 8.3%), and Pseudomonadota (5%) (Figure S2).

Members of the genus *Sedimentibacter* are known as rod-shaped, Gram-positive, amino acid- and pyruvate-utilizing, anaerobic bacteria, requiring yeast extract for growth and not utilizing carbohydrates [68]. Members of this genus have been detected in oilfields, in hydrocarbon-degrading enrichments [69,70], and in methanogenic enrichments from marine sediments [71]. It was suggested that bacteria of the genus *Sedimentibacter* act as syntrophs in such consortia. Other bacteria dominating in the enrichment belonged to the genus *Actinotalea* comprising aerobic or facultatively anaerobic organotrophic mesophilic bacteria [72,73,74,75,76]. Bacteria of the genera *Pseudomonas*, *Tepidimonas*, *Paeniclostridium*, *Rhodoferax*, and *Pusillimonas*, which often occur in subsurface environments, were among the minor components of the HO-5600 enrichment.

### 3.2. Isolation of Fermentative and Methanogenic Strains and Analysis of 16S rRNA Genes

**Figure 2:**
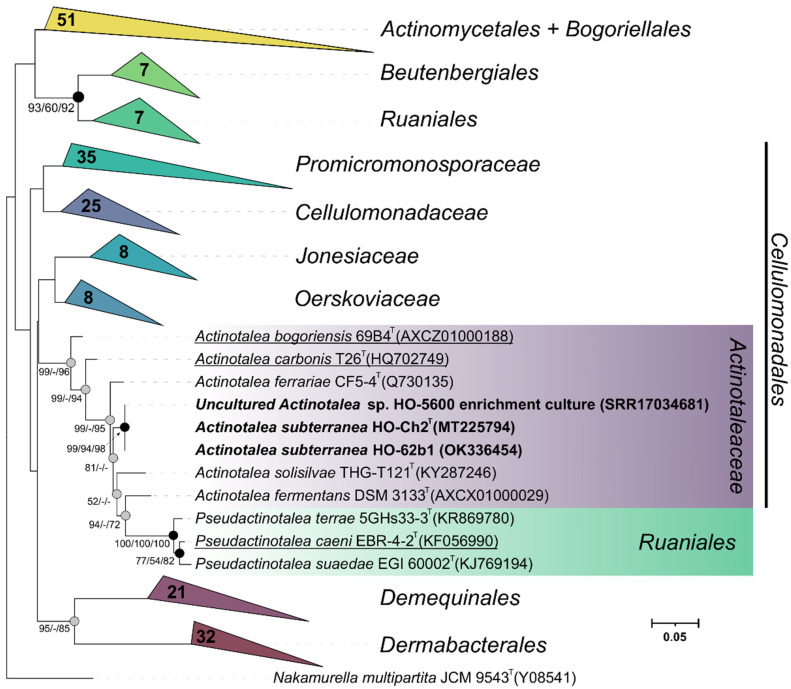
Maximum-likelihood phylogenetic tree based on 16S rRNA gene sequences (1462 nucleotide sites), showing the position of strains HO-Ch2^T^, HO-62b1 and the phylotype from the HO-5600 methanogenic enrichment among the closely related members of the class *Actinomycetia*. Gray circles indicate that the corresponding nodes were recovered in the tree reconstructed based on the maximum parsimony algorithm; black circles indicate that the corresponding nodes were also recovered based on the neighbor-joining and maximum-parsimony algorithms. Bootstrap values (>50%) are listed as percentages at the branching points. The tree was rooted using *Nakamurella multipartita* JCM 9543^T^ as the outgroup. GenBank accession numbers for the 16S rRNA gene sequences are indicated in parentheses. Bar, 0.05 substitutions per nucleotide position. Bacteria that have been reclassified are underlined.

Three strains were isolated in this study: the fermentative bacterial strain HO-Ch2^T^ and the methanogenic strain HO-Met1 isolated from the HO-5600 anaerobic methanogenic enrichment and aerobic strain HO-62b1 isolated from aerobic organotrophic enrichments. Strain HO-Met1 grew only on H_2_/CO_2_ with methane production. Based on the 99.9% sequence similarity of its 16S rRNA gene (GenBank accession number MT218393) with that of *Methanobacterium aarhusense* H2-LR^T^ [77], the new strain HO-Met1 was assigned to this species. The 16S rRNA gene sequences of strains HO-Ch2^T^ and HO-62b1 had 100% similarity with the zOTU of *Actinotalea* sp., determined from the HO-5600 methanogenic enrichment and could be assigned to one *Actinotalea* species. On the phylogenetic tree (Figure 2), these sequences formed a separate lineage within the genus *Actinotalea* with a high level of bootstrap support. Strain HO-Ch2^T^ was chosen for further in-depth study.

According to the EZBioCloud resource [37], the 16S rRNA gene of strain HO-Ch2T had high sequence similarity with those of *Actinotalea ferrariae* CF5-4^T^ (98.8%), *Actinotalea solisilvae* THG-T121^T^ (98.4%), and *Actinotalea fermentans* DSM 3133^T^ (97.6%) of the family *Actinotaleaceae* of the class *Actinomycetia* and could be assigned to the genus *Actinotalea*.

The 16S rRNA gene sequence similarity of 98.8% with *A. ferrariae* was slightly higher than 98.65%, the threshold accepted for species delineation [78]. Phylogenetic analysis of the 16S rRNA gene also revealed numerous discrepancies in the monophyletic nature of the genera within the family *Actinotaleaceae*. According to our results, type strains of the species *Cellulomonas carbonis* [79] and *Cellulomonas bogoriensis* [80] formed a cluster with members of the genus *Actinotalea*, which may indicate erroneous genus identification of these species. Moreover, the 16S rRNA gene sequences of *Actinotalea caeni* ERB-4-2^T^ clustered together with those of *Pseudactinotalea*, which indicated the probably required reclassification of this bacterium. It should be also noted that the branch uniting the *Pseudactinotalea* bacteria is anomalously long and may indicate incorrect position of this branching due to the Long Branch attraction [81] artifact. For elucidation of the taxonomic position of strain HO-Ch2^T^, elucidation of phylogenetic inconsistencies within the family *Actinotaleaceae*, and determination of its possible functional role in the oil-degrading community, morphological, physiological, and chemotaxonomic properties of the strain were studied, and its genome was sequenced and analyzed.

### 3.3. Phenotypic Characterization

Strains HO-Ch2^T^ and HO-62b1 and the closely related species, Actinotalea ferrariae CF5-4^T^, were phenotypically characterized. At the time of writing, the genus Actinotalea comprised four species: *A. fermentans*, *A. ferrariae*, *A. caeni*, and *A. solisilvae*, isolated from coal seams, iron mining powder, sludge of a biofilm reactor, and forest soil, respectively [72,73,74,75]. Recently, the genera *Actinotalea* and *Pseudactinotalea* were placed within the new family *Actinotaleaceae* of the order *Cellulomonadales* of the class *Actinomycetia* [76].

Cells of strains HO-Ch2^T^ and HO-62b1 were Gram-stain-positive rods, motile at the early stage of incubation. The cells of strains HO-Ch2^T^ were 0.4–0.7 µm wide and 1.4–2.6 µm long (Figure S3a,b). Both strains grew aerobically and anaerobically on R2A medium at 28 °C. Colonies formed after 5 days of growth on R2A medium at 28 °C were 1 mm in diameter, yellow, convex, circular, smooth and non-transparent, with entire edges. Strain HO-Ch2^T^ produced biofilms in liquid media (Figure S3c). The growth temperature range for the strain HO-Ch2^T^ was from 10 to 40 °C with optimal growth at 28 °C. The range of pH for growth was 6.0–8.8 with optimum pH 8.0–8.3; NaCl range was 0–4% (*w*/*v*) with optimum growth at 1–2% (*w*/*v*) NaCl (Figure S4a–c). Strain HO-62b1 grew at 15–40 °C (optimum 22–28 °C), at pH 6.2–8.5 (optimum pH 6.6–7.5), and with 0–6.0% (*w*/*v*) NaCl (optimum 2–4%) (Figure S4d–f). The strains were catalase-positive, oxidase-negative, and able to reduce nitrate to nitrite in the medium with acetate, but did not reduce nitrate to N_2_. Voges–Proskauer, indole and H_2_S production, and urease tests were negative. Growth of the strains under oxic and anoxic conditions (under mineral oil) was determined using API 50CH (Bio-Mérieux, France) tests; the results for both strains were completely identical. The rates of acidification of the medium by strains HO-Ch2^T^ and HO-62b1 under aerobic and anaerobic conditions were the same, while it was significantly higher for strain CF5-4^T^ grown under anaerobic conditions. Under aerobic conditions, strains HO-Ch2^T^ and HO-62b1 grew on PCA, R2A, TSB, and LB media, utilized peptone, yeast extract, acetate, and pyruvate; positive growth and acid production were observed with aesculin (Fe citrate), L-arabinose, arbutin, D-cellobiose, D-fructose, D-galactose, D-glucose, glycogen, D-mannose, salicin, D-sucrose, D-xylose, D-maltose, D-mannitol, starch, D-trehalose, D-turanose, and N-acetylglucosamine. Acid was not produced from D-adonitol, amygdalin, D-arabinose, D-arabite, L-arabite, dulcitol, erythritol, D-fucose, L-fucose, inositol, inulin, D-lactose, D-lyxose, methyl-βD-xylopyranoside, methyl-αD-mannopyranoside, methyl-αD-glucopyranoside, D-melibiose, D-melicitose, gluconate K, 2-ketogluconate K, 5-ketogluconate K, D-ribose, L-rhamnose, D-sorbitol, L-sorbose, D-tagatose, xylitol, and L-xylose (Table S1). In API 20E tests (Bio-Mérieux, France), strains HO-Ch2^T^, HO-62b1, and CF5-4^T^ were positive for fermentation/oxidation of glucose, sucrose, and arabinose; they were negative for arginine dihydrolase, lysine decarboxylase, ornithine decarboxylase, L-tryptophan deaminase, gelatinase, citrate utilization, indole production, and fermentation/oxidation of inositol, sorbitol, and rhamnose. New strains HO-Ch2^T^ and HO-62b1 differed from A. ferrariae CF5-4^T^ by their positive reaction for ß-galactosidase and fermentation/oxidation of mannitol, melibiose, and amygdalin (Table S2). In the API ^®^ ZYM tests (Bio-Mérieux, France), strain HO-Ch2^T^ was positive for esterase (C4), N-acetyl-β-glucosaminidase, α-galactosidase, β-galactosidase, α-glucosidase, β-glucosidase, β-glucuronidase, naphthol-AS-BI-phosphohydrolase, leucine arylamidase, esterase lipase (C8), lipase (C14), valine arylamidase, acid phosphatase, and cystine arylamidase activities, but negative for trypsin, α-chymotrypsin, alkaline phosphatase, α-mannosidase, and α-fucosidase (Table S3). Products of anaerobic glucose fermentation were acetic, propionic, iso-butyric, and iso-valeric acids and CO_2_. Hydrogen was not produced. Anaerobic growth of strain HO-Ch2^T^ in the MM medium with crude oil and 15 mM NaNO_3_ was studied. After 60 days of incubation at 28 °C slight changes in the C_16_–C_19_ alkane fraction (Figure S1d) and dimethylnaphthalenes content (data not shown) were revealed compared to those in the sterile control. The strain was sensitive to ampicillin (10 µg), chloramphenicol (30 µg), penicillin (10 µg), ciprofloxacin (5 µg), and erythromycin (15 µg), but resistant to gentamicin (10 µg) and kanamycin (30 µg). Physiological and biochemical characteristics of the strain HO-Ch2^T^ are summarized in the species description and selective characteristics distinguishing the strain from *Actinotalea* members are presented in Table 1.

### 3.4. Chemotaxonomic Characterization

The major cellular fatty acids (>5%) of strain HO-Ch2^T^ and the reference strain of *A. ferrariae* CF5-4^T^ were *anteiso*-C_15: 0_ (36.3 and 47.4%, respectively), C_16: 0_ (10.8 and 10.3%), C_14: 0_ (12.3 and 6.8%), C_15: 0_ (7.8 and 7.4%), and *anteiso*-C_15: 1_ (1.0 and 5.8%) (Table S4). The major polar lipids of strain HO-Ch2^T^ were diphosphatidylglycerols (DPG), unidentified glycolipids (GL3), and phosphoglycolipids (PGL2). Minor lipids included phosphatidylcholines (PC), phosphatidylglycerols (PG), four glycolipids (GL1, GL2, GL4, and GL5), phosphoglycolipids (PGL1), and two phospholipids (PL1, PL2) (Figures S5a and S6A). The major polar lipids of the reference strain *A. ferrariae* CF5-4^T^ included diphosphatidylglycerols (DPG), phosphatidylcholines (PC), and phosphoglycolipids (PGL4) (Figures S5b and S6B). Minor detected lipids of the strain CF5-4^T^ were five glycolipids (GL1-5), phosphatidylglycerols (PG), phosphoglycolipids (PGL1-3), and unidentified phospholipids (PL1 and PL2).

The respiratory quinones of strains HO-Ch2^T^ and *A. ferrariae* CF5-4^T^ contained MK-9(H_4_) as the major menaquinone, and MK-9(H_6_) and MK-9(H_2_) as the minor menaquinones at the ratios 10: 3: 1 and 10: 1.5: 0.5, respectively. The electron-impact mass spectrum of the isoprenoid quinone of strains HO-Ch2^T^ and CF5-4^T^ showed a base peak at m/z 211, and the peak of the molecular ion at m/z 789.4 (Figure S7). The corresponding values for MK-9(H_4_) were m/z 225 and 788, respectively. Traces of MK-8(H_4_) were also detected in both strains. Menaquinone MK-10(H_4_) has been found as a major menaquinone in the members of genus *Actinotalea*; however, *A. fermentans* DSM 3133^T^ contained MK-10(H_4_), MK-9(H_4_), and MK-8(H_4_) in the ratio 56: 2: 1 [72]. The spectrum of major menaquinones may vary among *Actinotalea* species, so that menaquinone composition may be unsuitable for accurate differentiation of members of the genus *Actinotalea* from *Cellulomonas* containing MK-9(H_4_). Amino acid analysis of the peptidoglycan preparation of HO-Ch2^T^ strain showed the presence of aspartic acid, serine, glutamic acid, alanine, and ornithine/lysin at an approximate molar ratio of 0.55: 2.0: 4.0: 6.1: 1.05/1.5, as well as glycin (5.8). The peptidoglycan of HO-Ch2^T^ strain corresponded to the A4β type containing L-Orn (Lys)-D-Ser-D-Glu. The molar ratio of ornithine, alanine, serine, D-glutamate, and aspartate in the peptidoglycan of *A. ferrariae* CF5-4^T^ was 0.8: 2.3: 0.9: 1.5: 1.1 (Table S5). Rhamnose was the major cell-wall sugar of strains HO-Ch2^T^ and CF5-4^T^ which is in accordance with features of the members of the genus *Actinotalea*. The composition of other polysaccharides varied and included galactose, mannose, and glucose in HO-Ch2^T^; and 3-O-methylgalactose (=madurosa), glucose, and traces of galactose and mannose were detected in *A. ferrariae* CF5-4^T^.

### 3.5. Whole Genome Sequencing and Phylogenomic Analysis

The final assembled 4,027,363-bp-long genome of the strain HO-Ch2T comprised 28 scaffolds, with an N50 value of 335,339 bp, and coverage of 197×. The genomic DNA G + C content of strain HO-Ch2T was 73.4%, which was slightly below the expected range reported for members of the genus *Actinotalea* (73.8–75.2%) (Table 2).

**Figure 3:**
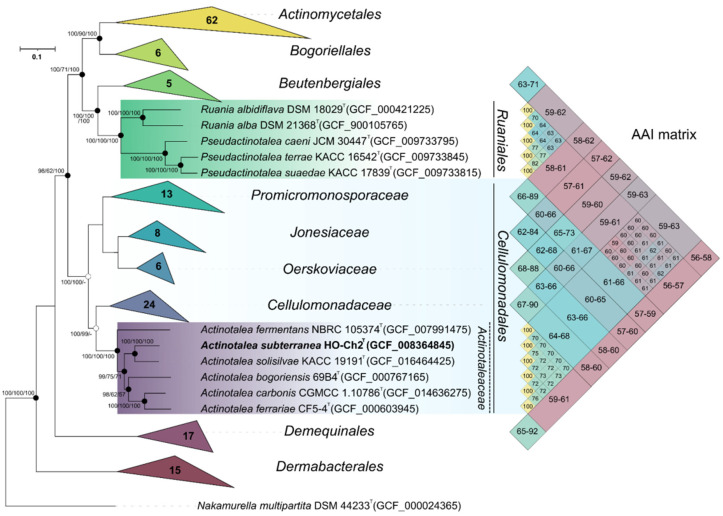
Maximum-likelihood phylogenetic tree derived from concatenated 120 single copy marker proteins showing the position of strain HO-Ch2^T^ in relation to taxonomically characterized members of the class *Actinomycetia*. Phylogenetic analysis was performed based on 34,747 amino acid positions. Bar, 0.1 amino acid substitutions per site. The tree was rooted using *Nakamurella multipartita* JCM 9543^T^ as the outgroup. Accession numbers for the genomic assemblies are indicated in brackets. The heatmap was build based on AAI values among representatives of the orders *Cellulomonadales*, *Ruaniales*, *Beutenbergiales*, and *Demequnales*.

The genome of the strain HO-Ch2^T^ contained 3678 genes, of which 3589 were protein-coding sequences, 38 were pseudogenes, and 51 were RNA genes. Functional annotation of the genome performed via the RASTtk pipeline revealed that 415 of the genes were associated with carbohydrate metabolism, 288 genes, with metabolism of amino acids and their derivatives, 224 genes, with protein metabolism, and 198 genes, with metabolism of cofactors, vitamins, and pigments (Figure S8). On the phylogenomic tree, strain HO-Ch2^T^ was placed within the genus *Actinotalea* (Figure 3). The ANI and dDDH values of 79.8–82.0% and 20.5–22.2%, respectively, to the *Actinotalea* genomes (Table 2) were below the species cutoff (95–96% for ANI and 70% for dDDH) [83], which indicated that the strain HO-Ch2^T^ belonged to a new species with proposed name *Actinotalea subterranea* sp. nov.

On the phylogenomic tree, as well as on the tree constructed using 16S rRNA gene alignment, type strains of *Cellulomonas carbonis* and *Cellulomonas bogoriensis* clustered together with members of the genus *Actinotalea*, which indicated the need for reclassification of these bacteria as members of the genus *Actinotalea*. Another confirmation of the need to reclassify these bacteria is the Average amino acid identity (AAI) values. *Cellulomonas carbonis* and *Cellulomonas bogoriensis* had AAI values with *Actinotalea* spp. in the range 69.7–76.3%, while only 65.4–67.7% with bacteria of the genus *Cellulomonas*. Based on phylogenetic analysis of the 16S rRNA gene, phylogenomic analysis and values of genomic indices, it was proposed to reclassify *Cellulomonas carbonis* and *Cellulomonas bogoriensis* as *Actinotalea carbonis* comb. nov. and *Actinotalea bogoriensis* comb. nov., respectively.

The phylogenomic tree confirmed the assumption that the taxonomic definition of *Actinotalea caeni* was incorrect. According to the results of phylogenetic studies and AAI values (Figure 3), this bacterium belongs to the genus *Pseudactinotalea* [84]. Moreover, despite their clustering on a 16S rRNA-based tree with bacteria of the genus *Actinotalea*, the phylogenomic tree indicates that the genus *Pseudactinotalea* belongs to the family *Ruaniaceae* [85]. The AAI values between *Pseudactinotalea* and *Ruania* are in the range of 63.1–64.2%, while between *Pseudactinotalea* and *Actinotalea* they are in the range 58.9–61.9%. Based on the above, it is proposed to reclassify *Actinotalea caeni* as *Pseudactinotalea caeni* comb. nov. and to transfer the genus *Pseudactinotalea* from the family *Actinotaleaceae* (*Cellulomonadales*) to the family *Ruaniaceae* of the order *Ruaniales*.

### 3.6. Pangenomic Analysis

**Figure 4:**
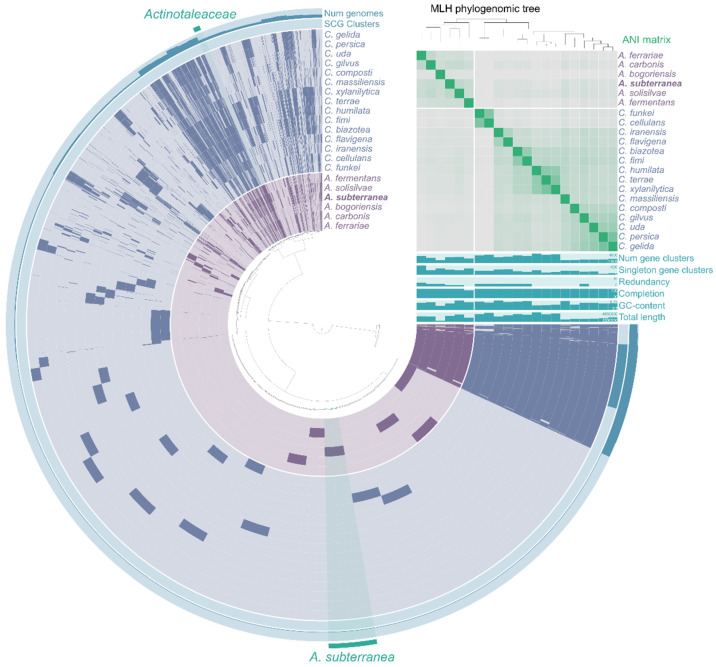
Pangenome analysis of *Actinotaleaceae* and *Cellulomonadaceae* calculated with Anvi’o versionv. 6.2. Dendrogram at the center represents the relationship between the 16,878 gene clusters (77,059 genes) found in analyzed genomes. Dark regions in colored circles represent genes found in that area for each genome. ANI heatmap in green squares vary between 70 and 100%. Phylogenomic tree reconstructed using the single copy genes.

A total of twenty-one genomes were used for the pangenomic analysis of *Actinotaleaceae* and *Cellulomonadaceae* species. The pangenome comprised 77,059 genes in 16,878 gene clusters (Figure 4). Of these 1137 gene clusters occurred in all bacterial genomes and were identified as the core ones for both families. Among these clusters were the genes responsible for complete pathways of carbohydrate metabolism: glycolysis (Embden-Meyerhof pathway), citrate cycle (TCA cycle), pentose phosphate cycle, UDP-N-acetyl-D-glucosamine biosynthesis. In addition, 32 gene clusters involved in energy metabolism (NADH:quinone oxidoreductase, succinate dehydrogenase, cytochrome bc1 complex, cytochrome c oxidase, F-type ATPase) were identified. All genomes of *Actinotaleaceae* and *Cellulomonadaceae* harbored also the nirBD nitrite reductase, which is responsible for the reduction of nitrite to ammonium.

The core genome of *Actinotaleaceae* comprised 1454 gene clusters, and 52 of them were unique to this family and mainly with hypothetical functions. All *Actinotaleaceae* genomes harbored the genes involved in sulfonate utilization (*ssuABCDE*), also sulfide:quinone oxidoreductase (*sqr*), and assimilatory nitrate reductase (*nasAB*). Strain HO-Ch2^T^ had 543 gene clusters unique for *Actinotaleaceae* and *Cellulomonadaceae*, and 106 of them were functionally annotated. Unique genes were involved in signaling and cellular processes (24), in metabolism of carbohydrates (16), amino acids (5), vitamins/cofactors (4), and energy (4). Unlike other bacteria of the *Actinotaleaceae* and *Cellulomonadaceae* families, strain HO-Ch2^T^ had in its genome the genes encoding anaerobic dimethyl sulfoxide reductase (*dmsAB*), L-rhamnonate dehydratase (*rhmD*), glucosamine kinase (*gspK*), propionate CoA-transferase (*pct*), and lycopene beta-cyclase (*crtY*).

## 4. Discussion

The biogeochemical processes of anaerobic transformation of oil with methane generation in reservoirs nor containing sulfate in waters or with hydrogen sulfide generation in reservoirs with sulfate-containing waters are well documented [1,7,10,86].

Degradation of oil *n*-alkanes has long been considered the process carried out mainly by members of the class *Deltaproteobacteria*, which use fumarate predominantly to activate alkane molecules [5,7,18]. Some members of this class, such as sulfate-reducing bacteria of the genera *Desulfatibacillum*, *Desulfosarcina/Desulfococcus*, *Desulfoglaeba*, and *Desulfatiferula*, grow with sulfide production in sulfate-containing environments on *n*-alkanes or *n*-alkenes as a sole carbon source [18]. Bacteria of the genera *Smithella* and *Syntrophus* degrade alkanes in consortia with methanogens [11]. Recently the archaeon ‘*Candidatus* Methanoliparum’ was shown to be able to combine the degradation of long-chain alkanes with methanogenesis [22].

In the present work, oil-degrading anaerobic methanogenic enrichment was obtained from a petroleum reservoir, its composition was determined by molecular 16S rRNA gene-based survey; methanogenic and fermentative components of the enrichment were isolated in pure cultures and taxonomically characterized.

Growth of the studied HO-5600 enrichment resulted in accumulation of H_2_ and methane in the gas phase and of acetate in the medium. The average rate of methane production by the studied culture for 275 days was 12.4 µmol·g of oil^−1^·day^−1^. This value was closed to the methane yield rate (2.9–8.8 µmol·g of oil^−1^·day^−1^) during heavy oil degradation by methanogenic consortium from the Shengli oil field, which preferentially degraded long-chain alkyl substituted hydrocarbons [87]. In this consortium, bacteria of the genera *Sedimentibacter*, *Soehngenia*, *Dehalococcoidetes*, *Actinobacteria*, *Anaerolineaceae*, *Clostridiales*, and unclassified bacteria and methanogenic archaea of the genera *Methanothrix*, *Methanosarcina* and of class *Methanomicrobia* were stable components of consortium persisting for 4 successive transfers.

The HO-5600 methanogenic enrichment harbored obligate anaerobes of the genera *Methanobacterium*, *Sedimentibacter*, and *Paeniclostridium* and aerobic or facultatively anaerobic organothrophic bacteria of the genera *Actinotalea*, *Pseudomonas*, *Tepidimonas*, and *Rhodoferax*. Using traditional cultivation techniques, two strains were isolated in pure cultures from a methanogenic enrichment maintained for a long time: strain HO-Met1, a hydrogenotrophic methanogen phylogenetically closed to *Methanobacterium aarhusense* (100% 16S rRNA sequence similarity), and strain HO-Ch2^T^, a facultatively anaerobic organothrophic bacterium belonging to the genus *Actinotalea*. While methanogenesis was the role *Methanobacterium aarhusense* strain HO-Met1 played in the enrichment [77], the roles of other components of the oil-degrading community remain unclear. Bacteria of the genera *Sedimentibacter* and *Actinotalea* predominated in the enrichment (49.1 and 26.8% of 16S rRNA gene sequences in the library, respectively). Some *Sedimentibacter* strains are known to utilize phenol, catechol, and benzoate, a central intermediate in several aromatic degradation pathways [68,71]. The major end products of amino acids utilization by *Sedimentibacter* sp. include acetate, propionate, and butyrate [71]. Anaerobic bacteria of the genus *Sedimentibacter* probably degraded the aromatic components of oil, releasing the products used by other members of the microbial community.

We should stress the predisposition of actinobacteria to oil-contaminated habitats. Bacteria of the genus *Actinotalea* were part of microbial biofilms (>1% of the community composition), degrading polycyclic aromatic hydrocarbons and naphthalene under microaerobic conditions [88]. *Actinotalea ferrariae* was the predominant component of the microbial community in the sample of oil-contaminated desert soil in some periods of the bioremediation process [89]. *Actinotalea* were numerous representatives of communities that degraded petroleum hydrocarbons in soil microbial fuel cells supplemented with biochar [90].

In our study, strain HO-62b1 identical to *Actinotalea* sp. strain HO-Ch2^T^ (100% similarity) was isolated from an aerobic organotrophic enrichment. On the phylogenetic tree (Figure 2), their sequences formed a separate lineage within the genus *Actinotalea* with the highest similarity (98.8%) to the sequences of the type strain of *Actinotalea ferrariae* CF5-4^T^. This value was slightly below the value 98.65% accepted for species delineation [75]. Physiology and phylogenetic position of strains HO-Ch2^T^ and HO-62b1 were determined, and the genome of strain HO-Ch2^T^ was sequenced for elucidation of their taxonomy and functioning in the oil-degrading community.

The results of phenotypic analysis of the facultatively anaerobic *Actinotalea* strains HO-Ch2^T^ and HO-62b1 revealed that they utilized a broad range of protein- and sugar-containing substrates under oxic and anoxic condition. Growth of both strains in the medium with crude oil was accompanied by slight changes in the C_16_–C_19_ alkane fraction and in dimethylnaphthalenes content compared to those in the sterile control. The probable ecophysiological function of these bacteria is fermentation of carbohydrate- and protein-containing substrates, including necromass (i.e., dead biomass). Fermentation of biomass by *Actinotalea* strains could results in generation of carbon sources (e.g., acetic, propionic, iso-butyric, and iso-valeric acids and CO_2_), supporting a microbial carbon turnover and recycling of other nutrients (e.g., N and P). Low-molecular-mass metabolic products of organotrophic bacteria are used by methanogenic members of the community, which carry out the terminal stage of oil biodegradation with release of methane.

Our conclusion correlates with results of previous study of facultative anaerobic enrichment cultures derived from the coal basins of eastern Australia [91]. Vik and co-workers isolated *Actinotalea* strain SUR-A1 from enrichment and based on phenotypic and genomics studies suggested potential metabolic and ecological roles of these bacteria in coal seams. It was believed that *Actinotalea* cannot directly participate in biodegradation of coal compounds in situ, but may be involved in the degradation of accumulated biomass in coal seams, providing with fermentation products other members of microbial community degrading coal to methane.

Results of the 16S rRNA gene sequence and core-genome analysis, the average nucleotide identity (ANI), and in silico DNA–DNA hybridization (dDDH), as well as the phenotypic and chemotaxonomic characterization supported the classification of strains HO-Ch2^T^ and HO-62b1 as belonging to a novel species of the genus *Actinotalea*, for which the name *Actinotalea subterranea* sp. nov. is proposed. Using the 16S rRNA gene sequences and genome sequences from the reference type strains of the class *Actinomycetia* from the GenBank database, the phylogenomic and pangenomic comparison was performed. These data elucidated the phylogenetic relationships among the genera *Actinotalea*, *Pseudactinotalea*, and *Cellulomonas* and confirmed the reclassification of *Cellulomonas carbonis* and *Cellulomonas bogoriensis* as new combinations within the genus *Actinotalea* and the transfer of the genus *Pseudactinotalea* to the family *Ruaniaceae* of the order *Ruaniales*.

## 5. Conclusions

Our data show that the anaerobic methanogenic enrichment obtained from a petroleum reservoir comprises anaerobic prokaryotes of the genera *Methanobacterium*, *Sedimentibacter*, and *Paeniclostridium* and facultatively anaerobic organothrophic bacteria of the genera *Actinotalea*, *Pseudomonas*, *Tepidimonas*, and *Rhodoferax*. Anaerobic growth on crude oil resulted in accumulation of H_2_ and CH_4_ in the gas phase and of acetate in the medium. Strain HO-Ch2^T^, isolated from the methanogenic enrichment, was capable of fermenting carbohydrate- and protein-containing components of microbial biomass with production of volatile fatty acids and CO_2_, and participated in carbon turnover in the oil-degrading enrichment. The polyphasic taxonomic study and phylogenomic analysis demonstrated that strain HO-Ch2^T^ constituted a novel species within the genus *Actinotalea*, for which the name *Actinotalea subterranea* sp. nov. is proposed. Genome analysis of the novel strain and of the closely related strains of the genera *Actinotalea*, *Pseudactinotalea*, and *Cellulomonas* supports the reclassification of *Cellulomonas carbonis* as *Actinotalea carbonis* comb. nov., *Cellulomonas bogoriensis* as *Actinotalea bogoriensis* comb. nov., *Actinotalea caeni* as *Pseudactinotalea caeni* comb. nov., and the transfer of the genus *Pseudactinotalea* to the family *Ruaniaceae* of the order *Ruaniales*. The taxonomic descriptions of the new species and new combinations are enclosed below.

### 5.1. Description of Actinotalea subterranea sp. nov.

*Actinotalea subterranea* (sub.ter.ra’nea. N.L. fam. adj. *subterranea* subterranean, because the organism grows naturally below the Earth’s surface).

Description is based on two strains. Cells are Gram-stain-positive rods, motile at the early stage of incubation. Colonies formed after 5 days incubation on R2A medium at 28 °C are yellow, smooth, circular, convex, and non-transparent, with entire edges. Grows on PCA, nutrient agar, R2A, and LB media. Growth of the type strain occurs in the presence of 0–4% (*w*/*v*) NaCl (optimum, 1–2% NaCl), at pH 6.0–8.8 (optimum, pH 8.0–8.3) and at 10–40 °C (optimum, 28 °C). Growth of the reference strain occurs at 15–40 °C, 0–6% (*w*/*v*) NaCl and pH 6.2–8.5 with optimal conditions at 22–28 °C, 2–4% (*w*/*v*) NaCl and pH 6.6–7.5. Catalase-positive and oxidase-negative. Cells are positive for the following enzyme activities: esterase (C4), N-acetyl-β-glucosaminidase, α-galactosidase, β-galactosidase, α-glucosidase, β-glucosidase, β-glucuronidase, naphthol-AS-BI-phosphohydrolase, leucine arylamidase, esterase lipase (C8), lipase (C14), valine arylamidase, acid phosphatase, and cystine arylamidase, but negative for trypsin, α-chymotrypsin, alkaline phosphatase, α-mannosidase, urease, and α-fucosidase. Does not produce indole or H_2_S, but NH_3_ is produced from peptone. Negative for the methyl red test and Voges–Proskauer reaction. Reduces nitrate to nitrite in a medium with acetate, but does not reduce nitrate to N_2_. Chemoorganoheterotrophic, facultatively anaerobic. In aerobic conditions utilizes peptone, yeast extract, acetate, and pyruvate; acid is produced from aesculin (Fe citrate), L-arabinose, arbutin, D-cellobiose, D-fructose, D-galactose, D-glucose, glycogen, D-mannose, salicin, D-sucrose, D-xylose, D-maltose, D-mannitol, starch, D-trehalose, D-turanose, and N-acetylglucosamine, but not from D-adonitol, amygdalin, D-arabinose, D-arabite, L-arabite, dulcitol, erythritol, D-fucose, L-fucose, inositol, inulin, D-lactose, D-lyxose, methyl-βD-xylopyranoside, methyl-αD-mannopyranoside, methyl-αD-glucopyranoside, D-melibiose, D-melicitose, gluconate K, 2-ketogluconate K, 5-ketogluconate K, D-ribose, L-rhamnose, D-sorbitol, L-sorbose, D-tagatose, xylitol, and L-xylose. In API 20E tests, positive for fermentation/oxidation of glucose, sucrose, and arabinose; and negative for arginine dihydrolase, lysine decarboxylase, ornithine decarboxylase, L-tryptophan deaminase, gelatinase, citrate utilization, indole production, and fermentation/oxidation of inositol, sorbitol, and rhamnose. Acetic, propionic, iso-butyric, and iso-valeric acids and CO_2_ are the major products of anaerobic glucose fermentation. Hydrogen is not produced. Sensitive to ampicillin (10 µg), chloramphenicol (30 µg), penicillin (10 µg), ciprofloxacin (5 µg), and erythromycin (15 µg), but resistant to gentamicin (10 µg) and kanamycin (30 µg). The peptidoglycan type is A4β, containing L-Orn (Lys)-D-Ser-D-Glu. The major cell-wall sugar is rhamnose; galactose, mannose, and glucose are also present. Major fatty acids (>5%) are *anteiso*-C_15:0_, C_14:0_, C_16:0_, and C_15:0_. The major menaquinone is MK-9(H_4_). The major polar lipids are diphosphatidylglycerol, unidentified glycolipids, and phosphoglycolipids.

The type strain is HO-Ch2^T^ (= VKM Ac-2850^T^ = KCTC 49656^T^), isolated from the methanogenic enrichment obtained from a petroleum reservoir (Nurlat, Russia). The DNA G + C content of the genome of the type strain HO-Ch2^T^ is 73.4% and the genome size is 4.0 Mb. The EMBL/GenBank accession numbers for the 16S rRNA gene sequence and genome sequence of strain HO-Ch2^T^ are MT225794 and GCA_008364845.1, respectively.

### 5.2. Description of Actinotalea carbonis, comb. nov.

*Actinotalea* *carbonis* (car.bo’nis. N.L. fem. adj. *carbonis* isolated from coal mine soil, charcoal).

Basonym: *Cellulomonas carbonis* Shi et al. 2012 emend. Nouioui et al. 2018.

The description is as given for *Cellulomonas carbonis* (Shi et al., 2012; Nouioui et al., 2018). The type strain is T26 (= CGMCC 1.10786 = KCTC 19,824 = CCTCC AB2010450).

### 5.3. Description of Actinotalea bogoriensis, comb. nov.

*Actinotalea* *bogoriensis* (bo.go.ri.en’sis. N.L. fem. adj. *bogoriensis* pertaining to Lake Bogoria, Kenya).

Basonym: *Cellulomonas bogoriensis* Jones et al. 2005.

The description is as given for *Cellulomonas bogoriensis* (Jones et al., 2005) with the following modification. The G+C content of the type-strain genome is 72.2%, its approximate size 3.18 Mbp, and its GenBank deposit SAMN03113094. The type strain is 69B4 (= DSM 16,987 = CIP 108683).

### 5.4. Emended description of the family Actinotaleaceae Salam et al. 2020

The description is as given for *Actinotaleaceae* (Salam et al., 2020) with the following modification. This family contains *Actinotalea* (Yi et al. 2007 emend. Yan et al. 2018), which is the type and currently the sole genus of the family.

### 5.5. Description of Pseudactinotalea caeni, comb. nov.

*Actinotalea caeni* (cae’ni. L. gen. n. *caeni* of sludge).

Basonym: *Actinotalea caeni* Jin et al. 2017.

The description is as given for *Pseudactinotalea caeni* (Jin et al., 2017) with the following modification. The genomic G+C of the type-strain is 73.4%, and its approximate size 4.00 Mbp, its GenBank deposit SAMN13336169. The type strain is EBR-4-2 (=KCTC 33604 = JCM 30447).

### 5.6. Emended Description of the Genus Pseudactinotalea Cho et al. 2017 Emend. Salam et al. 2020

*Pseudactinotalea* (Pseud.ac.ti.no.ta’le.a. Gr. adj. *pseudes* false; *Actinotalea* a bacterial genus name; N.L. fem. n. *Pseudactinotalea* a false *Actinotalea*).

The description is as given before (Salam et al., 2020) with the following modification. The genomic G+C content is around 70–74%. Genome sizes vary from 4.00 to 4.75 Mb. The type species is *Pseudactinotalea terrae.* The genus *Pseudactinotalea* is phylogenetically positioned within the family *Ruaniaceae* (Tang et al., 2010), order *Ruaniales* (Salam et al., 2020), class *Actinomycetia* of the phylum Actinobacteria.

## Figures and Tables

**Table 1:** Characteristics differentiating strain HO-Ch2^T^ from *Actinotalea* species *.

Characteristic	HO-Ch2^T^	*A. ferrariae* CF5-4^T^	*A. fermentans* M^T^ [72,82]	*A. solisilvae* THG-T121^T^ [75]	*A. carbonis* T26^T^ [79]	*A. bogoriensis* 69B4^T^ [80]
Motility	+	–	–	–	+	+
Temperature range (optimum), °C	10–40 (28)	4–40 (28)	10–40 (30–37)	10–40 (28–30)	4–45 (28)	20–37 (30–37)
pH range (optimum)	6–9 (7.5–8.5)	6–8 (7)	6–11 ^a^	6–8 (7)	6.0–10 (7)	6.0–10.5 (9–10)
NaCl range (optimum), %, *w*/*v*	0–4.5 (1)	0–7 (3)	0–7 (2–4)	0–4 (1)	0–7	0–8
Catalase	+	+	–	+	+	+
Oxidase	–	–	+	+	–	
Hydrolysis of:						
Chitin	+	–	– ^a^	– ^a^	ND	ND
Gelatin	–	+	+	+	+	+
Nitrate reduction to NO_2_^−^	+	+	+	–	+	–
Activity and assimilation of (API 20E or API 50CH)						
Acetate	+	+	+	+	–	–
N-Acetylglucosamine	+	–	– ^a^	+	+	–
Gluconate	–	–	–	+	+	–
Lactate	–	–	–	+	–	–
Propionate	–	–	–	+	+	ND
D-Galactose	+	+	+	ND	+	–
D-Glucose	+	+	+	–	+	+
Lactose	–	–	+	ND	+	–
Melibiose	–	–	–	+	+	–
Raffinose	W	–	+	ND	W	–
D-Ribose	–	+	–	+	–	–
L-Rhamnose	–	–	–	+	–	+
Inositol	–	–	+ ^a^	+	–	–
Glycerol	W	+	–	ND	ND	–
D-Mannitol	+	+	+	–	–	–
D-Sorbitol	–	–	+ ^a^	+	+	–
Enzyme activity: (API ZYM)						
Acid phosphatase	–	+	– ^a^	+	+	ND
Alkaline phosphatase	–	+	– ^a^	+	–	–
Esterase lipase (C8)	+	+	– ^a^	+	–	+
Valine arylamidase	+	+	– ^a^	–	–	ND
Cystine arylamidase	+	+	– ^a^	+	–	ND
N-acetyl-β-glucosaminidase	+	+	– ^a^	+	+	ND
α-Galactosidase	+	+	– ^a^	–	+	ND
β-Galactosidase	+	–	+	+	+	ND
Lipase (C14)	+	–	–^a^	–	–	ND
Major fatty acids	ai-C_15:0_, C_14: 0_, C_16:0_, C_15:0_	ai-C_15:0_, C_16:0_, C_15:0_	C_14:0_, ai-C_15:0_, C_16:0_	ai-C_15:0_, ai-C_15:1_ A, C_16:0_	ai -C_15:0_, ai -C_15:1_ A, C_16:0_	ai -C_15:0_, C_16:0_, C_14: 0_
Peptidoglycan ^1^	L-Orn (Lys)-D-Ser-D-Glu	L-Orn–D-Ser–D-Asp	L-Orn-D-Ser-L-Asp^b^	L–Orn–D–Ser–L–Asp	L-Orn– D-Glu	L-Orn–D-Asp
Cell–wall sugars ^2^	Rha, Gal, Man, Glc	Rha, Fuc, Man, Gal^b^	Rha, Rib, Glc	Rha, Rib, Man, Glc	Rha, Gal, Xyl, Ino	ND
Major polar lipids ^3^	DPG, GL, PGL, PC, PG, PL	DPG, PC, PGL, PG, GL, PL	DPG, PG^c^	DPG, PG, PI, PIM, PL, GL, L	DPG, PG, PIM, PI, PL, PGL	DPG, PG, PIM, PI, PL, PGL^b^
Quinones	MK-9(H_4_), MK-9(H_6_), MK-8(H_4_)	MK–10(H_4_)	MK–10(H_4_), MK–9(H_4_), MK–8(H_4_)	MK–10(H_4_)	MK–9(H_4_)	MK-9(H_4_)
Isolation source	Methanogenic enrichment from the oil field	Iron mining powder	Methanogenic enrichment from dumping ground	Forest soil	Coal mine soil	Sediment of the littoral zone of the lake

Positive results for all strains are obtained for: activity of leucine arylamidase, naphtol-AS-BI-phosphohydrolase, esterase (C4), β-Galactosidase, α-glucosidase, β-Glucosidase; hydrolysis of aesculin, cellulose, and starch; assimilation of salicin, L-arabinose, cellobiose, maltose, and sucrose. Negative for: indole production test and activity of arginine dihydrolase. Designations: +, Positive; –, negative; w, weakly positive; ND, no data available. * Data for strains HO-Ch2^T^ and CF5-4^T^ are from this study, except as labeled. ^a^, Data from Yan et al. [75]; ^b^, Data from Li et al. [73]; ^c^*,* Data from Shi et al. [79]. ^1^ Orn, ornithine; Ser, serine; Asp, aspartatic acid; Glu, glutamic acid. ^2^ Fuc, fucose; Gal, galactose; Glc, glucose; Ino, inositol; Man, mannose; Rha, rhamnose; Rib, ribose; Xyl, xylose. ^3^ DPG, diphosphatidylglycerols; PG, phosphatidylglycerols; PI, phosphatidylinositols; PIM, phosphatidylinositol mannosides; PL, unidentified phospholipids; GL, unidentified glycolipids; L, unidentified lipids; PC, phosphatidylcholines.

**Table 2:** General properties and relationship of the genomes between strain HO-Ch2^T^ and type strains of the genus *Actinotalea*.

Attribute	HO-Ch2^T^	*A. ferrariae* CF5-4 ^T^	*A. solisilvae* KACC 19191^T^	*A. fermentans* DSM 3133^T^	*A. carbonis* T26^T^	*A. bogoriensis* 69B4^T^
Genome size (bp)	4,027,363	3,987,077	4,296,322	3,737,518	3,904,075	3,183,361
G+C content (%)	73.4	73.8	75.2	74.1	74.2	72.2
DNA scaffolds	28	626	41	22	73	524
Total genes	3678	3907	3957	3450	3527	3241
Protein coding genes	3589	3697	3851	3369	3433	3015
Number of tRNA	45	45	46	45	45	45
Number of rRNA	3	8	5	3	3	4
Pseudo genes	38	154	51	30	43	174
ANI (%)	100	80.2	82.0	80.1	81.0	79.8
dDDH (%)	100	21.4	22.2	20.5	21.5	20.5

## Data Availability

The library of 16S rRNA gene fragments of the HO-5600 methanogenic enrichment culture was deposited in NCBI SRA, project no. SRR17034681. The GenBank/EMBL/DDBJ accession numbers of the 16S rRNA gene sequence of strains HO-Ch2^T^, HO-62b1, and HO-Met1 are MT225794, OK336454, and MT218393, respectively. The whole-genome shotgun project of strain HO-Ch2^T^ has been deposited at DDBJ/EMBL/GenBank under the accession VTTP00000000.1, and it is the first version described in this paper. The raw FASTQ reads have been deposited in the NCBI SRA database under the accession no. SRR10092497.

## Supplementary Materials

The following supporting information can be downloaded at: https://www.mdpi.com/2076-2607/10/2/378/s1. Figure S1: Chromatograms of the saturated hydrocarbon fraction of sterile oil (a) and oil degraded by the HO-5600 enrichment (b); residual content of n-alkanes (%) in oil degraded by the HO-5600 methanogenic enrichment (c) and by strain HO-Ch2^T^ (d) after 2 months incubation with 0.5% (*v*/*v*) crude oil under anaerobic conditions at 28 °C. Medium MM for strain HO-Ch2^T^ was amended with 15 mM NaNO_3_; Figure S2: The relative proportion of the 16S rRNA gene fragment sequences of Bacteria and Archaea represented at the genus level in the library from the HO-5600 anaerobic enrichment after 36 weeks of incubation with crude oil. The taxa comprising > 1% in the library are shown; Figure S3: Scanning electron micrograph (a) and transmission electron micrograph (b) of strain HO-Ch2^T^ cells and biofilms (c) grown in MS medium with yeast extract (1 g·l^−1^) at 28 °C for 72 h. The images were obtained under a Camscan-S2 scanning electron microscope (Cambridge, UK) (accelerating voltage 20 kV, SEI mode) (a) and under a JEM-100C transmission electron microscope (JEOL, Japan) (b). Bars, 1 µm (a, b); Figure S4: Growth profiles of strains HO-Ch2^T^ (a-c) and HO-62b1 (d-f) at various temperatures (a, d), pH (b, e) and NaCl concentrations (*w*/*v*, %) (c,f); Figure S5: Two dimensional thin layer chromatogram of polar lipids from strain HO-Ch2^T^ (a) and *Actinotalea ferrariae* CF5-4^T^ (b). The components were visualized by staining with 5% sulfuric acid in ethanol and heating at 180 °C for 15 min. Abbreviations: DPG, diphosphatidylglycerols; PC, phosphatidylcholines; PG, phosphatidylglycerols; PL1–PL2, unidentified phospholipids; GL1–GL5, unidentified glycolipids; PGL1–PGL4, unidentified phosphoglycolipids; Figure S6: Identification of the polar lipids from strain HO-Ch2^T^ (A) and *A. ferrariae* CF5-4^T^ (B). The components were visualized by molybdenum blue for phospholipids (a); α-naphthol for glycolipids (b); ninhydrin for aminolipids (c); Dragendorff’s reagent for choline-containing lipids (d). Abbreviations: PC, phosphatidylcholine; PL1–PL9, unidentified phospholipids; GL1–GL9, unidentified glycolipids; Figure S7: Mass-spectrum of menaquinones from strain HO-Ch2^T^ (a) and *A. ferrariae* CF5-4^T^ (b) showing the presence of menaquinones MK-9(H_4_) (peak 789.4), MK-9(H_2_) (peak 787), MK-9(H_6_) (peak 791), and traces of MK-8(H_4_) (peak 721); Figure S8: Subsystems in the genome sequence of strain HO-Ch2^T^ based on SEED database; Table S1: Carbohydrate fermentation by strains HO-Ch2^T^, HO-62b1, and *A. ferrariae* CF5-4^T^ determined by the API 50CH test (bioMérieux, France). +, positive; –, negative; Table S2: Comparison of enzymatic activities of strains HO-Ch2^T^, HO-62b1, and *A. ferrariae* CF5-4^T^ determined by the API 20E test (bioMérieux, France). +, positive; –, negative; Table S3: Comparison of enzymatic activities of strains HO-Ch2^T^, HO-62b1, and *A. ferrariae* CF5-4^T^ determined by the API ^®^ ZYM test (bioMérieux, France). +, positive; –, negative; W, weakly positive; Table S4: Cellular fatty acid composition* of strain HO-Ch2^T^ and type strain of *A. ferrariae* CF5-4^T^; Table S5: Amino acid composition of cell walls of strains HO-Ch2^T^ and *A. ferrariae* CF5-4^T^.
