# Correction: Semenova et al. Physiological and Genomic Characterization of *Actinotalea subterranea* sp. nov. from Oil-Degrading Methanogenic Enrichment and Reclassification of the Family *Actinotaleaceae. Microorganisms* 2022, *10,* 378

**DOI:** 10.3390/microorganisms10050862

**Published:** 2022-04-21

**Authors:** Ekaterina M. Semenova, Denis S. Grouzdev, Diyana S. Sokolova, Tatiyana P. Tourova, Andrey B. Poltaraus, Natalia V. Potekhina, Polina N. Shishina, Maria A. Bolshakova, Alexander N. Avtukh, Elena A. Ianutsevich, Vera M. Tereshina, Tamara N. Nazina

**Affiliations:** 1 Winogradsky Institute of Microbiology, Research Center of Biotechnology, Russian Academy of Sciences, 119071 Moscow, Russia; semenova_inmi@mail.ru (E.M.S.); sokolovadiyana@gmail.com (D.S.S.); tptour@rambler.ru (T.P.T.); e.a.ianutsevich@gmail.com (E.A.I.); v.m.tereshina@inbox.ru (V.M.T.); 2 SciBear OU, 10115 Tallinn, Estonia; denisgrouzdev@gmail.com; 3 Engelhardt Institute of Molecular Biology, Russian Academy of Sciences, 119991 Moscow, Russia; abpolt@gmail.com; 4 Faculty of Biology, Lomonosov Moscow State University, 119991 Moscow, Russia; potekhina56@mail.ru; 5 Geological Faculty, Lomonosov Moscow State University, 119991 Moscow, Russia; shishina_p@mail.ru (P.N.S.); m.bolshakova@oilmsu.ru (M.A.B.); 6 Skryabin Institute of Biochemistry and Physiology of Microorganisms, Pushchino Scientific Center for Biological Research, Russian Academy of Sciences, Pushchino, 142290 Moscow, Russia; avtukh@rambler.ru

The authors wish to make the following correction to this paper [1].

The authors note that the KCTC number of the type strain HO-Ch2^T^ appeared incorrectly in Section 5.1. *Description of Actinotalea subterranea sp. nov.*—in the published version of the paper. The correct version of the KCTC number of the type strain HO-Ch2^T^ is as follows:

HO-Ch2^T^ (= VKM Ac-2850^T^ = KCTC 49656^T^).

The authors apologize for any inconvenience caused and state that the scientific conclusions are unaffected. The original publication has also been updated.
