# Supplementary material for: Physiological and Genomic Characterization of Actinotalea subterranea sp. nov. from Oil-Degrading Methanogenic Enrichment and Reclassification of the Family Actinotaleaceae
Source: Microorganisms. 2022 Feb 6;10(2):378. doi: 10.3390/microorganisms10020378 (PMC8878594; doi:10.3390/microorganisms10020378)
Supplement: Supplementary file 1 [file microorganisms-10-00378-s001.zip › Supplementary Materials-1560480.pdf]

microorganisms

MDPI

Supplementary Materials

Physiological and Genomic Characterization of *Actinotalea subterranea* sp. nov. from Oil-Degrading Methanogenic Enrichment and Reclassification of the Family *Actinotaleaceae*

Ekaterina M. Semenova [1], Denis S. Grouzdev [2], Diyana S. Sokolova [1], Tatiyana P. Tourova [1], Andrey B. Poltaraus [3], Natalia V. Potekhina [4], Polina N. Shishina [5], Maria A. Bolshakova [5], Alexander N. Avtukh [6], Elena A. Ianutsevich [1], Vera M. Tereshina [1] and Tamara N. Nazina [1,*]

[1] Winogradsky Institute of Microbiology, Research Center of Biotechnology of the Russian Academy of Sciences, 119071 Moscow, Russia; semenova_inmi@mail.ru (E.M.S.); sokolovadiyana@gmail.com (D.S.S.); tptour@rambler.ru (T.P.T.); e.a.ianutsevich@gmail.com (E.A.I.); v.m.tereshina@inbox.ru (V.M.T.); nazina@inmi.ru (T.N.N.)

[2] SciBear OU, 10115 Tallinn, Estonia; denisgrouzdev@gmail.com (D.S.G.)

[3] Engelhardt Institute of Molecular Biology, Russian Academy of Sciences, 119991 Moscow, Russia; abpolt@gmail.com (A.B.P.)

[4] Lomonosov Moscow State University, Faculty of Biology, 119991 Moscow, Russia; potekhina56@mail.ru (N.V.P.)

[5] Lomonosov Moscow State University, Geological Faculty, 119991 Moscow, Russia; shishina_p@mail.ru (P.N.S.), m.bolshakova@oilmsu.ru (M.A.B.)

[6] Skryabin Institute of Biochemistry and Physiology of Microorganisms, Russian Academy of Sciences, Pushchino Scientific Center for Biological Research of the Russian Academy of Sciences, Pushchino, Moscow region 142290, Russia; avtukh@rambler.ru (A.N.A.)

* Correspondence: nazina@inmi.ru; Tel.: +7-499-135-0341

Chemotaxonomic Characterization of Membrane Lipids, Peptidoglycan and Sugars

Membrane lipids were analyzed as described earlier [59]. Wet biomass was homogenized in 2-propanol, and incubated at 70 °C for 30 min. Thereupon, the lipids were extracted using the method described by Nichols [60] with a few changes, which involved extraction with 2-propanol and a 2-propanol/chloroform mixture (1 : 1 and 1 : 2) at 70 °C, evaporation in a rotary evaporator, and extraction of the residue with chloroform/methanol (1 : 1) supplemented with 5% sodium chloride solution and water to remove water-soluble substances. After separating the mixture by allowing it to stand overnight, the chloroform layer was dried by passing it through water-free sodium sulfate, evaporated, and desiccated with a vacuum pump. The resulting pellet was dissolved in chloroform/methanol (2 : 1, v/v) and stored at –21 °C. Phospholipids and glycolipids were separated on glass plates with silica gel 60 (Merck, Germany) using the following solvent systems for two-dimensional TLC: chloroform/methanol/water (65 : 25 : 4, by vol.) in the first dimension and chloroform/acetone/methanol/acetic acid/water (50 : 20 : 10 : 10 : 5, by vol.) in the second dimension [61]. The lipids (100–200 μg) were applied to a plate. To develop the stains, the chromatograms were sprayed with 5% sulfuric acid in ethanol, with subsequent heating to 180 °C. Phospholipids were identified using individual markers and several spraying reagents for visualizing the spots: molybdenum blue for phospholipids [62], ninhydrin for amino lipids, α-naphthol for glycolipids and the Dragendorff's reagent for choline-containing phospholipids [63].

Peptidoglycan and sugars in the whole cell-wall of strains HO-Ch2T and *A. ferrariae* CF5-4^{T} were analyzed as described in [64]. Native cell walls of the strains were obtained by differential centrifugation after preliminary disruption by sonication (UP100H, Hielscher, Germany, 30 kHz, 3–5 times per 10 min) and purified with 2% (w/v) sodium dodecyl sulfate (SDS). To obtain peptidoglycan, carbohydrate-containing polymers were removed from the cell wall (30 mg) by extraction with 5% TCA at 100 °C for 20 min. Trypsin solution (1 mg / ml Tris-HCl buffer, pH 7.85) was added (10 ml) to the remains of the cell walls, and enzyme treatment was carried out at 37 °C for 20 h. The obtained preparation was repeatedly washed with distilled water and then treated with 4% (w/v) SDS in the same buffer at 100 °C for 5 min. After washing from SDS, the peptidoglycan preparation was lyophilized. Amino acids of peptidoglycan were determined after sample hydrolysis (freshly prepared concentrated hydrochloric and trifluoroacetic acids in a 2 : 1 ratio with the addition of 0.1% β-mercaptoethanol, 1 h at 155 °C) with L-8800 amino acid analyzer (Hitachi, Tokyo, Japan) according to the method described in [65]. The products of acid hydrolysis (2M HCl, 3 h, 100 °C) of the cell wall preparations was identified by paper chromatography using solvent systems (pyridine–benzene–butan-1-ol–H_2O (3 : 1 : 5 : 3, v/v) and specific reagents ($AgNO_3$, aniline phthalate) as described in [64].

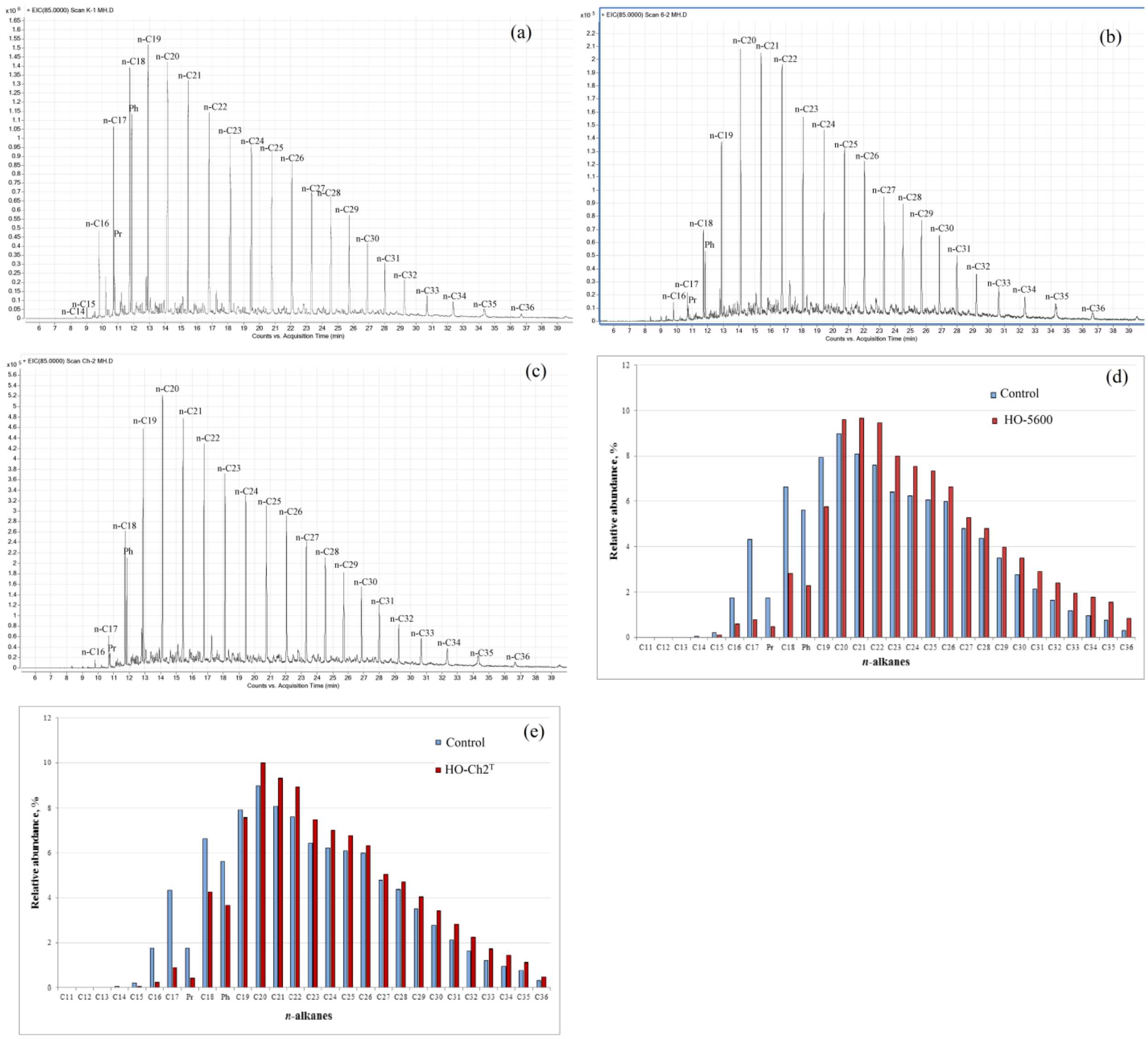

Figure S1. Chromatograms of the saturated hydrocarbon fraction of sterile oil (a) and oil degraded by the HO-5600 enrichment (b) and strain HO-Ch2^T(c); relative abundance of *n*-alkanes (%) in oil degraded by the HO-5600 methanogenic enrichment (d) and by strain HO-Ch2^T (e) after 1 year incubation with 0.5% (vol/vol) crude oil under anaerobic conditions at 28 °C. Medium MM for strain HO-Ch2^T was amended with 15 mM $NaNO_3$.

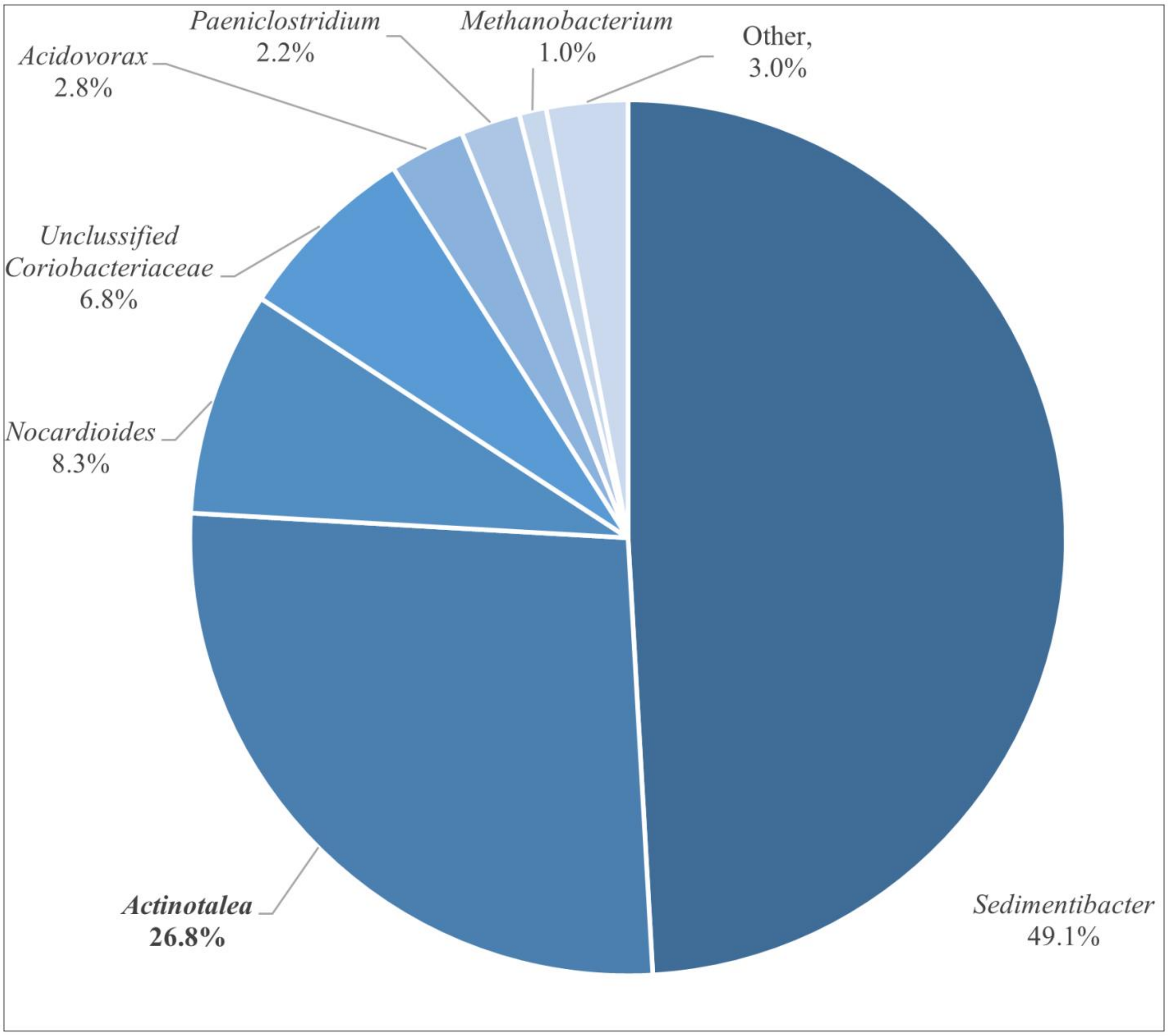

Figure S2. The relative proportion of the 16S rRNA gene fragments sequences of *Bacteria* and *Archaea* represented at the genus level in the library from the HO-5600 anaerobic enrichment after 36 weeks of incubation with crude oil. The taxa comprising > 1% in the library are shown.

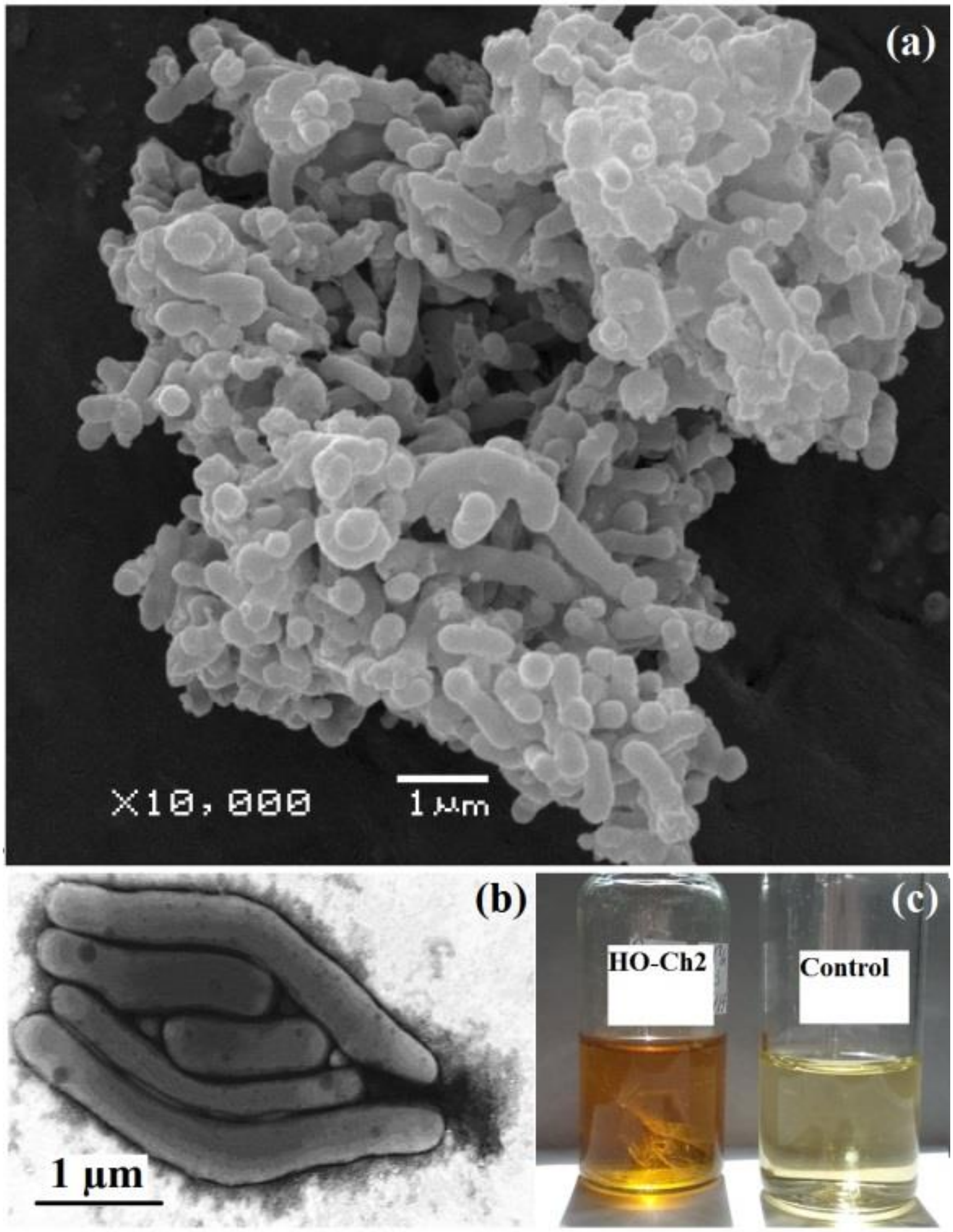

Figure S3. Scanning electron micrograph (a) and transmission electron micrograph (b) of strain HO-Ch2^T cells and biofilms (c) grown in MS medium with yeast extract (1 g·l^{-1}) at 28 °C for 72 h. The images were obtained under a scanning electron microscope Camscan-S2 (Cambridge, UK) (accelerating voltage 20 kV, SEI mode) (a) and under a JEM-100C transmission electron microscope (JEOL, Japan) (b). Bars, 1 μm (a, b).

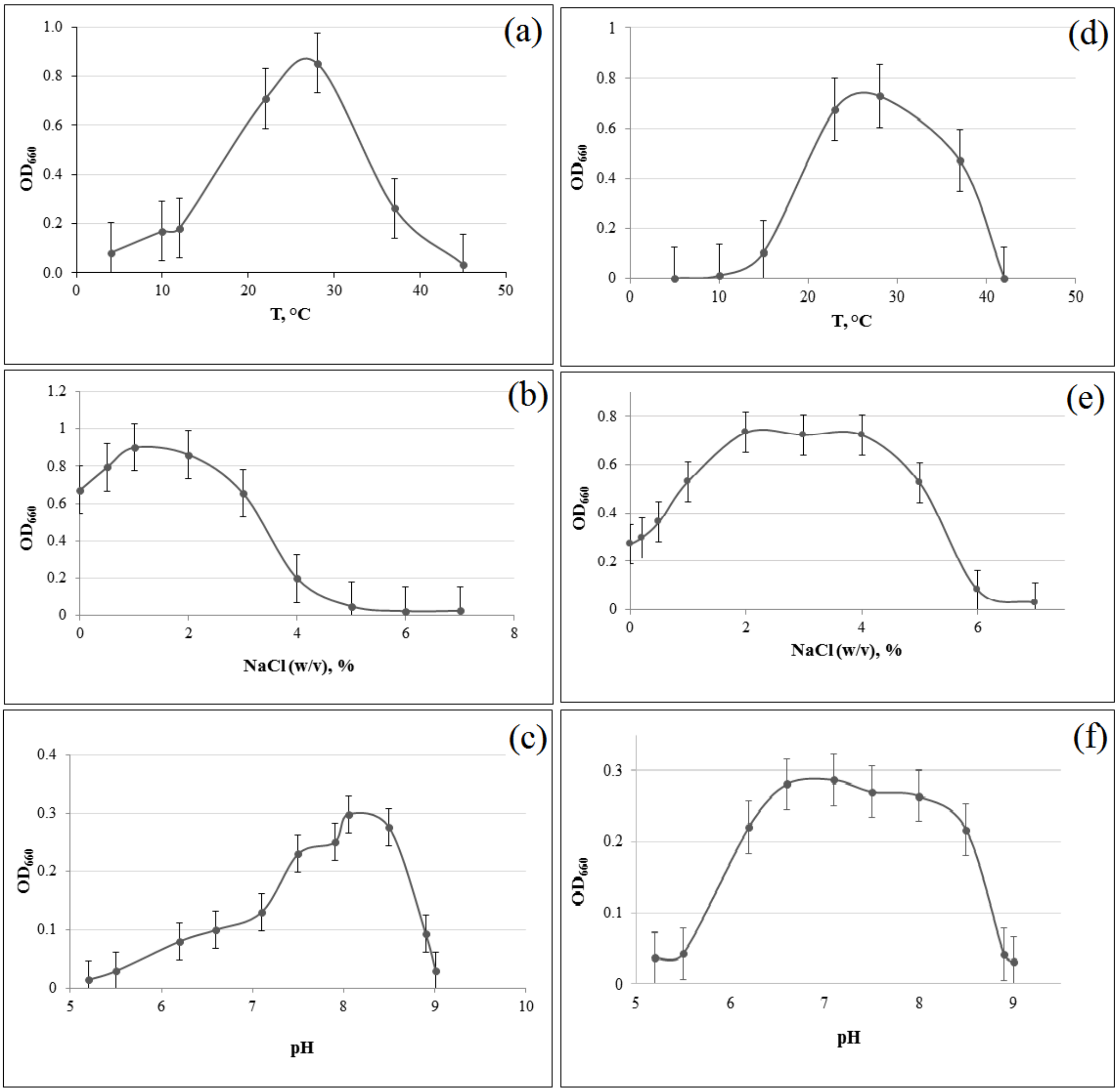

Figure S4. Growth profiles of strains HO-Ch2T (a-c) and HO-62b1 (d-f) at various temperatures (a, d), pH (b, e) and NaCl concentration (w/v, %) (c, f).

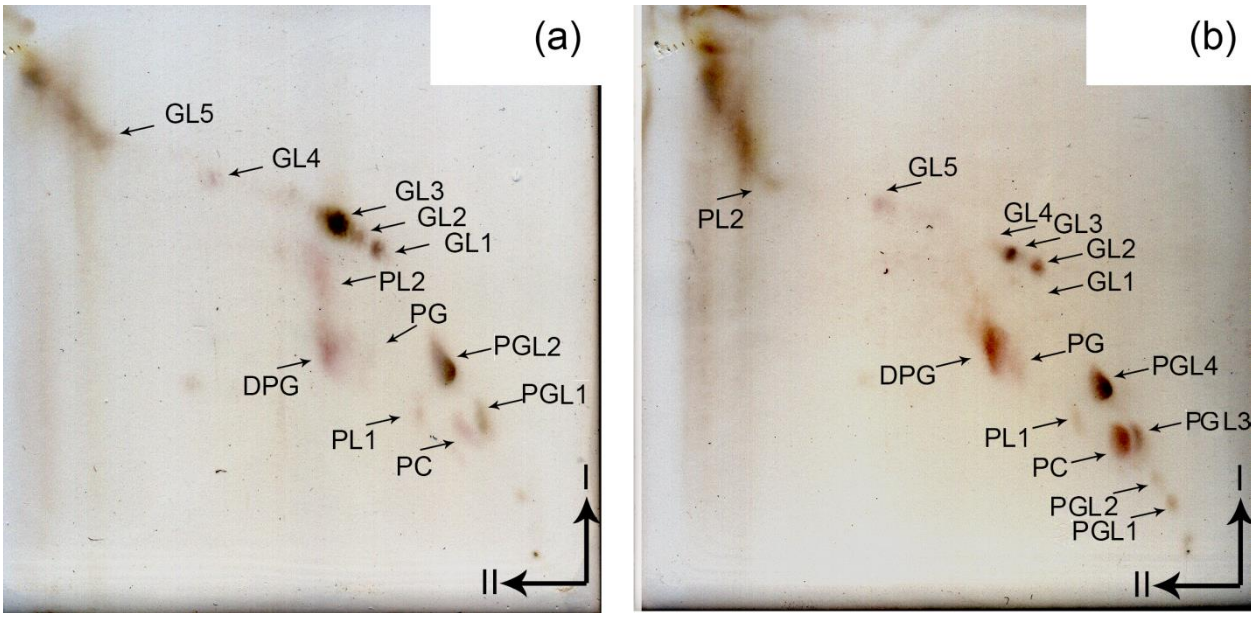

Figure S5. Two-dimensional thin layer chromatogram of polar lipids from strain HO-Ch2^T (a) and *Actinotalea ferrariae* CF5-4^T (b). The components were visualized by staining with 5% sulfuric acid in ethanol and heating at 180 °C for 15 min. Abbreviations: DPG, diphosphatidylglycerols; PC, phosphatidylcholines; PG, phosphatidylglycerols; PL1–PL2, unidentified phospholipids; GL1–GL5, unidentified glycolipids; PGL1–PGL4, unidentified phosphoglycolipids.

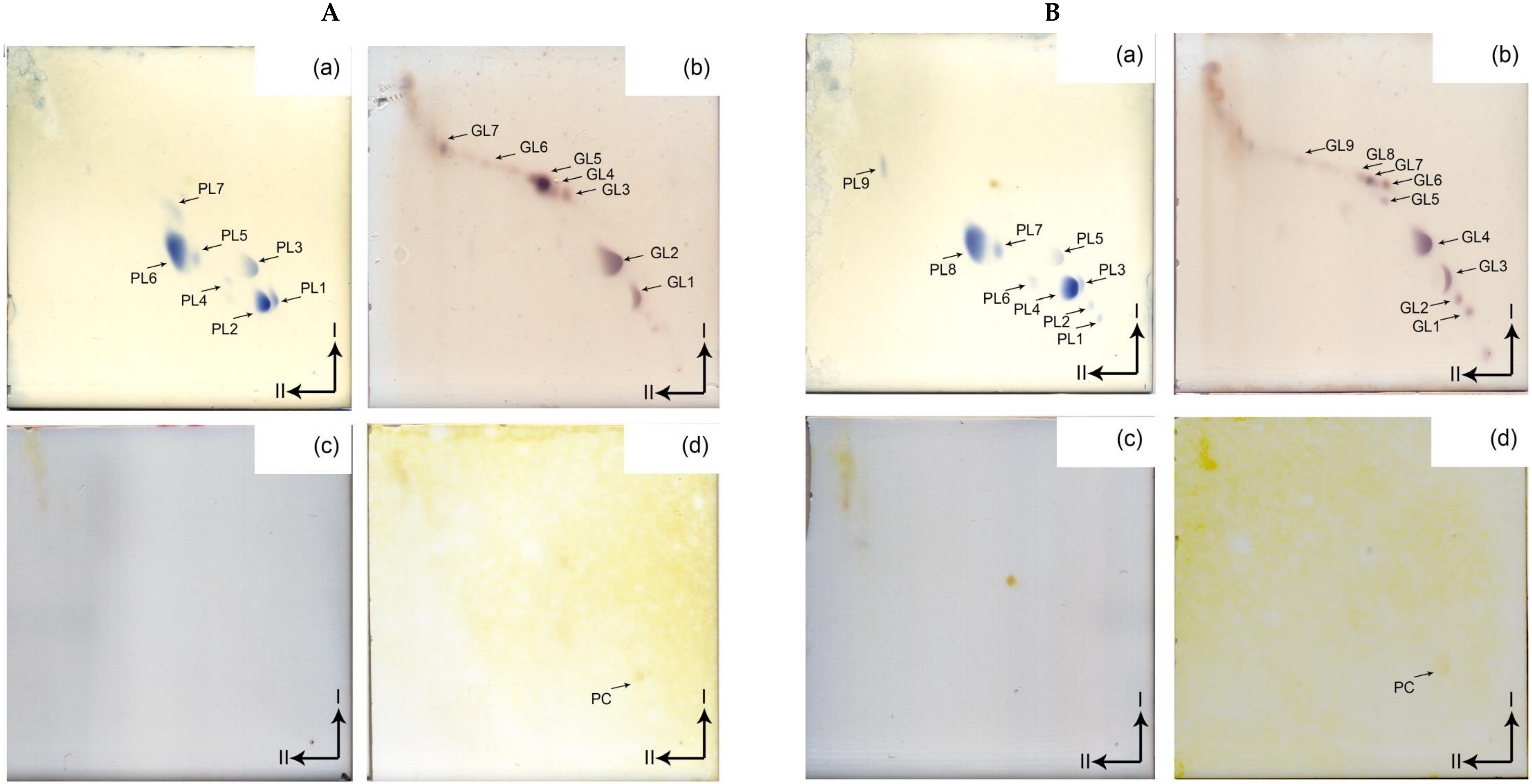

Figure S6. Identification of the polar lipids from strain HO-Ch2^T (A) and *A. ferrariae* CF5-4^T (B). The components were visualized by molybdenum blue for phospholipids (a); α-naphthol for glycolipids (b); ninhydrin for aminolipids (c); Dragendorff's reagent for choline-containing lipids (d). Abbreviations: PC, phosphatidylcholine; PL1–PL9, unidentified phospholipids; GL1–GL9, unidentified glycolipids.

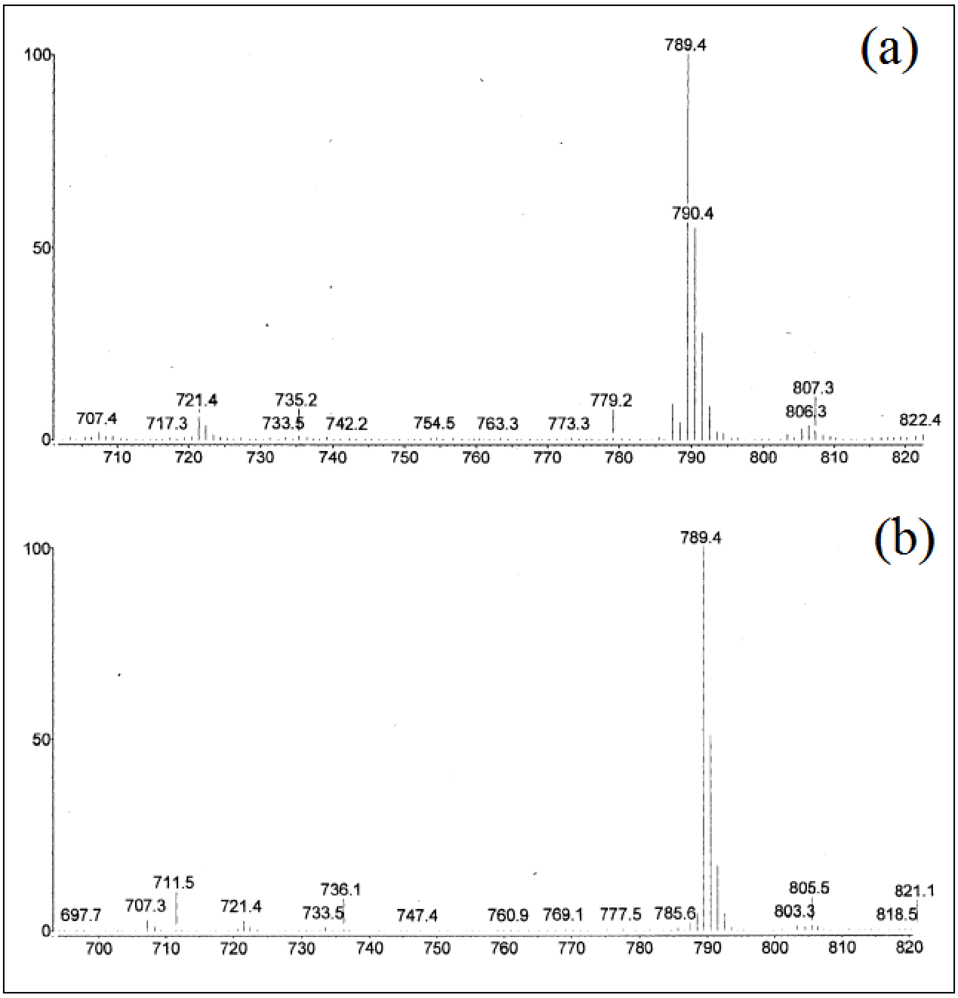

Figure S7. Mass-spectrum of menaquinones from strain HO-Ch2[T] (a) and *A. ferrariae* CF5-4[T] (b) showing the presence of menaquinones MK-9(H_4) (peak 789.4), MK-9(H_2) (peak 787), MK-9(H_6) (peak 791), and traces of MK-8(H_4) (peak 721).

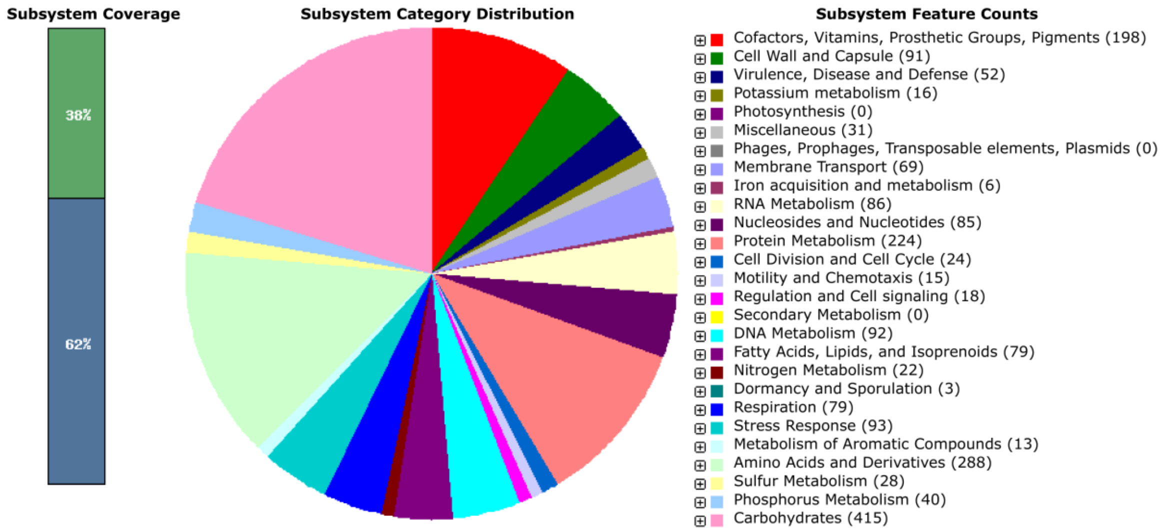

Figure S8. Subsystems in the genome sequence of strain HO-Ch2[T] based on SEED database.

Table S1. Carbohydrate fermentation by strains HO-Ch2^T, HO-62b1, and *A. ferrariae* CF5-4^T determined by the API 50CH test (bioMérieux, France). +, positive; –, negative.

No.	Substrate	HO-Ch2^T	HO-62b1	CF5-4^T
0	Control	–	–	–
1	Glycerol	+/-	+/-	+
2	Erythritol	–	–	–
3	D-Arabinose	–	–	–
4	L-Arabinose	+	+	+
5	D-Ribose	–	–	+
6	D-Xylose	+	+	+
7	L-Xylose	–	–	–
8	D-Adonitol	–	–	–
9	Methyl-βD-xylopyranoside	–	–	–
10	D-Galactose	+	+	+
11	D-Glucose	+	+	+
12	D-Fructose	+	+	+
13	D-Mannose	+	+	+
14	L-Sorbose	–	–	–
15	L-Rhamnose	–	–	–
16	Dulcitol	–	–	–
17	Inositol	–	–	–
18	D-Mannitol	+	+	–*
19	D-Sorbitol	–	–	–
20	Methyl-αD-mannopyranoside	–	–	–
21	Methyl-αD-glucopyranoside	–	–	+
22	N-acetylglucosamine	+	+	–
23	Amygdalin	–	–	–
24	Arbutin	+	+	–
25	Aesculin (Fe citrate)	+	+	+
26	Salicin	+	+	+
27	D-Cellobiose	+	+	+
28	D-Maltose	+	+	+
29	D-Lactose	–	–	–
30	D- Melibiose	–	–	–
31	D-Sucrose	+	+	+
32	D-Trehalose	+	+	+
33	Inulin	–	–	–
34	D-Melicitose	–	–	–
35	D-Raffinose	+	–	–
36	Starch	+	+	+
37	Glycogen	+	+	+
38	Xylitol	–	–	–
39	Gentiobiose	+	+	–
40	D-Turanose	+	+	+
41	D-lyxose	–	–	–
42	D-Tagatose	–	–	–
43	D-Fucose	–	–	–
44	L-Fucose	–	–	–
45	D-Arabite	–	–	–
46	L-Arabite	–	–	–
47	Gluconate K	–	–	–
48	2-Ketogluconate K	–	–	–
49	5-Ketogluconate K	–	–	–

*These data are positive in the paper Li et al. [18].

Table S2. Comparison of enzymatic activities of strains HO-Ch2^T, HO-62b1, and *A. ferrariae* CF5-4^T determined by the API 20E test (bioMérieux, France). +, positive; –, negative.

REACTIONS	HO-Ch2^T	HO-62b1	CF5-4^T
ß-Galactosidase	+	+	–
Arginine dihydrolase	–	–	–
Lysine decarboxylase	–	–	–
Ornithine decarboxylase	–	–	–
Citrate utilization	–	–	–
H_2S production	–	–	–
Urease	–	–	–
Tryptophane deaminase	–	–	–
Indole production	–	–	–
Acetoin production (Voges Proskauer)	–	–	–
Gelatinase	–	–	–
Fermentation / oxidation (glucose)	+	+	+
Fermentation / oxidation (mannitol)	+	+	–
Fermentation / oxidation (inositol)	–	–	–
Fermentation / oxidation (sorbitol)	–	–	–
Fermentation / oxidation (rhamnose)	–	–	–
Fermentation / oxidation (saccharose)	+	+	+
Fermentation / oxidation (melibiose)	+	+	–
Fermentation / oxidation (amygdalin)	+	+	–
Fermentation / oxidation (arabinose)	+	+	+
$NO_3^- \rightarrow NO_2^-$	+	+	+

Table S3. Comparison of enzymatic activities of strains HO-Ch2^T, HO-62b1, and *A. ferrariae* CF5-4^T determined by the API®ZYM test (bioMérieux, France). +, positive; –, negative; W, weakly positive.

Enzyme	HO-Ch2^T	HO-62b-1	CF5-4^T
Control	–	–	–
Alkaline phosphatase	–	–	+
Esterase (C4)	+	+	+
Esterase Lipase (C8)	+	+	+
Lipase (C14)	+	–	–
Leucine arylamidase	+	+	+
Valine arylamidase	+	+	+
Cystine arylamidase	+	+	+*
Trypsin	–	–	–
α-Chymotrypsin	–	–	–
Acid phosphatase	+	+	+
Naphthol-AS-BI-phosphohydrolase	+	+	+
α-Galactosidase	+	+	+
ß-Galactosidase	+	+	–
ß-Glucuronidase	+	–	–
α-Glucosidase	+	+	+*
ß-Glucosidase	+	+	+*
N-acetyl-ß-glucosaminidase	+	+	+
α-Mannosidase	–	–	–
α-Fucosidase	–	–	– (beta)

*These data are negative in the paper Li et al. [18].

Table S4. Cellular fatty acid compositions* of strain HO-Ch2^T and type strain of *A. ferrariae* CF5-4^T.

Fatty acid	HO-Ch2^T	CF5-4^T
$C_{12:0}$	0.51	0.33
iso-$C_{13:0}$	0.40	–
anteiso-$C_{13:0}$	2.39	1.32
iso-$C_{14:0}$	4.62	3.25
$C_{14:0}$	**12.34**	6.85
anteiso-$C_{15:1}$	1.04	5.80
iso-$C_{15:0}$	1.55	2.91
anteiso-$C_{15:0}$	**36.35**	**47.40**
$C_{15:0}$	**7.82**	**7.45**
iso-$C_{16:0}$	1.26	2.02
$C_{16:0}$	**10.82**	**10.32**
$C_{17:1}$	3.65	–
anteiso-$C_{17:0}$	2.72	3.85
$C_{17:0}$	1.60	–
$C_{18:2}\omega 9,12$	2.48	1.69
$C_{18:1}\omega 9$	3.71	3.49
$C_{18:0}$	4.19	3.08
Others	2.55	0.24
	100.00	100.00

*The values are percentages (w/w) of total fatty acids. Dominant fatty acids are indicated in bold.

Table S5. Amino acid composition of cell walls of strains HO-Ch2^T and *A. ferrariae* CF5-4^T.

Strain	Molar ratio of amino acids§							
	**Asp**	**Ser**	**Glu**	**Ala**	**Orn**	**Gly**	**OH-Lys**	**Lys**
HO-Ch2^T	0.55	2.0	4.0	6.09	1.05	5.8	Traces	1.5
A. ferrariae CF5-4^T	1.1	0.88	1.53	2.3	0.84	0.42	0.41	Traces

§Asp, aspartatic acid; Ser, serine; Glu, glutamic acid; Ala, alanine; Orn, ornithine; Gly, glycin; OH-Lys, Lys, lysin.
